# Prediction of benign paroxysmal positional vertigo recurrence in postmenopausal women: a machine learning-based clinical study

**DOI:** 10.3389/fneur.2026.1890770

**Published:** 2026-08-25

**Authors:** Xueqin Mi, Xuemeng Xu, Lei Fan, Qingchun Pan

**Affiliations:** 1 Department of Otolaryngology, Chengdu Sixth People's Hospital, Chengdu, China; 2 Sichuan Provincial Bayi Rehabilitation Center, Chengdu, China; 3 Department of Otolaryngology, North Sichuan Medical College Affiliated Hospital, Nanchong, China

**Keywords:** benign paroxysmal positional vertigo, feature selection, machine learning, postmenopausal women, predictive model

## Abstract

**Background:**

Benign paroxysmal positional vertigo (BPPV) is a common peripheral vestibular disorder with a high recurrence rate that significantly impairs patients’ quality of life. Multiple studies have confirmed that postmenopausal women, due to endocrine disorders and other physiological characteristics, represent a high-risk population for both BPPV onset and recurrence. However, efficient recurrence prediction models are currently lacking, and traditional assessment methods have limited efficacy, making it difficult to meet the needs of individualized intervention. The aim of this study was to construct and validate machine learning models for predicting BPPV recurrence in postmenopausal women, identify key risk factors, and provide evidence for early identification of high-risk populations in clinical practice.

**Methods:**

We retrospectively analyzed data from BPPV patients diagnosed and successfully treated at three hospitals in Sichuan Province between January 2023 and December 2025. Patients were divided into training and validation sets at a 7:3 ratio. Features were screened using LASSO regression and the Boruta algorithm. Six machine learning models were constructed: Logistic Regression (LR), Extreme Gradient Boosting (XGBoost), Support Vector Machine (SVM), Multilayer Perceptron (MLP), Gradient Boosting Machine (GBM), and Meta-Ensemble method. Model predictive performance was compared, and model interpretability was assessed through SHapley Additive exPlanations (SHAP) analysis.

**Results:**

Through dual algorithmic screening, LASSO, and Boruta delineated four model-selected predictors for BPPV recurrence in postmenopausal women: osteoporosis, serum calcium, vitamin D, and estradiol. Among the six machine learning algorithms evaluated, the Meta-Ensemble model yielded the highest AUC point estimate of 0.857 (95% confidence interval [CI], 0.779–0.928), delivering a harmonized overall performance. Subsequent SHAP analysis elucidated that decreased serum levels of vitamin D, calcium, and estradiol, compounded by the presence of osteoporosis, were the most critical factors driving high recurrence risk predictions.

**Conclusion:**

The Meta-Ensemble model can effectively predict BPPV recurrence risk in postmenopausal women. The four indicators of osteoporosis, serum calcium, vitamin D, and estradiol provide objective evidence for early clinical identification of high-risk populations for recurrence, facilitating the development of individualized follow-up strategies and intervention protocols.

## 1 Introduction

Benign paroxysmal positional vertigo (BPPV) is the most common peripheral vestibular disorder, with a lifetime prevalence of approximately 10% (1). Although canalith repositioning procedures are effective for acute symptom control, recurrence remains a major clinical challenge: the one-year recurrence rate ranges from 15 to 30%, and the 10-year cumulative recurrence rate reaches as high as 50% (2, 3). Repeated episodes significantly increase patients’ risks of falls, fractures, and anxiety and depression, while causing repeated consumption of medical resources (4, 5). Postmenopausal women represent the highest-risk population for BPPV recurrence, with an incidence approximately 2–3 times that of men of the same age, and the peak onset age closely overlaps with the natural menopausal age range (50–70 years) (6). Therefore, establishing a recurrence risk prediction tool targeted at this specific population is of practical significance for improving clinical management.

Although recent studies have begun to develop predictive models for BPPV recurrence in general populations (7), and specifically for postmenopausal women using traditional logistic regression (8), there remains a lack of models that leverage advanced machine learning to capture the complex, nonlinear interactions among metabolic and hormonal factors in this specific subgroup. On one hand, risk factor screening predominantly relies on univariate or multivariate Logistic regression (9, 10), whose linear framework struggles to capture the nonlinear interactions among calcium metabolism, hormonal regulation, and bone homeostasis in BPPV pathophysiology. On the other hand, predictive models are mostly based on traditional regression methods; although Logistic regression nomograms have been reported (8), systematic comparisons of multiple machine learning models are lacking, particularly regarding the application of ensemble learning and meta-ensemble strategies in predicting BPPV recurrence in postmenopausal women.

Based on the above background, this study focuses on BPPV recurrence in postmenopausal women, employing LASSO regression combined with the Boruta algorithm for feature screening, systematically comparing the predictive performance of six machine learning models including Logistic Regression, SVM, MLP, GBM, XGBoost, and Meta-Ensemble, and utilizing SHAP analysis to achieve model interpretation, with the aim of providing a risk assessment method that combines predictive accuracy and interpretability for clinical practice, assisting in the early identification of high-risk patients after canalith repositioning and targeted secondary prevention.

## 2 Methods

### 2.1 Study population

A total of 412 BPPV patients admitted to Chengdu Sixth People’s Hospital, North Sichuan Medical College Affiliated Hospital, and Sichuan Bayi Rehabilitation Center between January 2023 and December 2025 were enrolled. Patients were randomly divided into a training set (*n* = 289) and a validation set (*n* = 123) at a 7:3 ratio using a computer-generated random number method with a fixed random seed [set.seed(42)]. To ensure balanced representation of recurrence status, stratified random sampling was employed. Baseline characteristics between the training and validation cohorts were compared, confirming good comparability between the two groups (Supplementary Table 1).

Inclusion criteria: (1) met the diagnostic criteria for BPPV (11); (2) no abnormalities on MRI or cranial CT examination; (3) all were postmenopausal women. Postmenopausal status was strictly defined as the absence of menstruation for at least 12 consecutive months. Consistent with clinical endocrine definitions, this included natural menopause, premature ovarian insufficiency (before age 40), and medically/surgically induced menopause (e.g., bilateral oophorectomy or chemotherapy). Exclusion criteria: (1) concomitant autoimmune diseases; (2) concomitant hepatic or renal dysfunction; (3) presence of malignant tumors; (4) concomitant hypothyroidism or hyperthyroidism; (5) vertigo caused by sudden sensorineural hearing loss, vestibular neuritis, head trauma, brain tumors, stroke, or central demyelinating diseases; (6) concomitant psychiatric disorders.

Recurrence criteria: BPPV recurrence was defined as the reappearance of typical positional vertigo symptoms within 6 months after successful canalith repositioning, confirmed as positive by the Dix-Hallpike test or Roll test. Follow-up method: All patients were enrolled for follow-up after successful repositioning, through a combination of telephone follow-up and outpatient review, with follow-up continuing until December 2025.

This study was approved by the Ethics Committee of North Sichuan Medical College Affiliated Hospital (2020ER035-1), and patients and their families provided informed consent for the study.

### 2.2 Observation indicators

Outcome variable: BPPV recurrence (yes/no).

Study variables: This study collected the following 20 clinical indicators from patients: age, years of education, residence, body mass index (BMI), otolith position, Dizziness Handicap Inventory (DHI) score, Hospital Anxiety and Depression Scale anxiety subscale (HADS-A) and depression subscale (HADS-D), Pittsburgh Sleep Quality Index (PSQI), as well as estradiol, calcium, and 25(OH)D levels, while also recording histories of hypertension, hyperlipidemia, diabetes, migraine, cervical spondylosis, Meniere’s disease, osteoporosis, and head trauma.

The specific assessment criteria for each indicator were as follows:

Dizziness Handicap Inventory (DHI): Used to assess dizziness-related functional disability, with a total score ranging from 0 to 100 points, graded as follows: 0–30 points = mild; 31–60 points = moderate; 61–100 points = severe.

Hospital Anxiety and Depression Scale (HADS): Contains two subscales for anxiety (HADS-A) and depression (HADS-D), each with 7 items and a total score of 0–21 points. Scoring criteria: 0–7 points = normal; 8–10 points = mildly abnormal; ≥11 points = abnormal. A score of ≥8 on either subscale indicates possible anxiety or depressive symptoms (12).

Pittsburgh Sleep Quality Index (PSQI): Used to assess sleep quality; a score ≥8 indicates poor sleep quality, and <8 indicates good sleep quality (13).

Bone mineral density and osteoporosis assessment: Lumbar spine (L1–L4) bone mineral density was measured using a Hologic whole-body dual-energy X-ray absorptiometry (DXA) scanner. The T-score was calculated as: T-score = (measured BMD—mean peak BMD of healthy young adults of the same sex)/standard deviation of young adult BMD. T-score >−1.0 = normal bone mass; T-score between −1.0 and −2.5 = osteopenia; T-score ≤−2.5 = osteoporosis.

Biochemical index detection: After diagnosis of BPPV and successful repositioning, all patients had venous blood collected in the morning (08:00–10:00) to minimize diurnal variation in 25(OH)D levels. During the study period (January 2023–June 2024), patients were recruited with stratification by season to balance seasonal effects. After centrifugation, 25(OH)D and estradiol concentrations were measured using an immunofluorescence analysis system; calcium levels were measured using a semi-automatic analyzer. 25(OH)D: <20 ng/mL = deficient; 20–30 ng/mL = insufficient; >30 ng/mL = sufficient (based on Endocrine Society Clinical Practice Guidelines). Calcium: 2.25–2.75 mmol/L = normal; <2.25 mmol/L = abnormal (14).

Hypertension: Defined according to the Chinese Clinical Practice Guidelines for Hypertension (2022 Edition) (15) as systolic blood pressure ≥140 mmHg and/or diastolic blood pressure ≥90 mmHg.

Hyperlipidemia: According to the Chinese Guidelines for the Prevention and Treatment of Dyslipidemia in Adults (2016 Revised Edition) (16), the diagnostic criteria were fasting triglycerides ≥1.7 mmol/L or total cholesterol ≥5.72 mmol/L.

Diabetes: According to the Chinese Type 2 Diabetes Prevention and Treatment Guidelines (2020 Edition) (17), defined as fasting blood glucose ≥7.0 mmol/L or 2-h postprandial blood glucose ≥11.1 mmol/L.

### 2.3 Sample size estimation

Based on a reported BPPV recurrence rate of approximately 20% in previous studies, with an expected AUC of 0.80, significance level alpha = 0.05, and statistical power 1-beta = 0.80, the minimum required sample size was calculated to be 320 cases. Considering a 10% dropout rate, a total of 412 patients were enrolled, meeting the sample size requirement.

### 2.4 Feature selection for machine learning

The overall dataset was randomly divided into training and validation sets at a 7:3 ratio. Prior to modeling, missing data were evaluated; missing values were minimal (<5% for any variable) and were imputed using median (for continuous variables) or mode (for categorical variables) imputation. To strictly prevent data leakage, the imputation parameters and feature scaling (standardization) were fitted exclusively on the training set and subsequently applied to the validation set. Crucially, feature selection was also properly restricted to the training data: LASSO regression (with the optimal penalty coefficient lambda determined through 10-fold cross-validation, using the 1se criterion) and the Boruta algorithm (based on random forest importance assessment) were applied solely to the training set, and the intersection of both methods was taken as the final predictive features. These selected features were then fixed and applied to the validation set. Spearman correlation analysis was used to assess collinearity among features. All preprocessing steps were integrated into the machine learning pipeline to ensure independent estimation within each cross-validation fold.

### 2.5 Machine learning model construction and performance evaluation

This study constructed six machine learning models based on the screened features: Logistic Regression (LR), Extreme Gradient Boosting (XGBoost), Support Vector Machine (SVM), Multilayer Perceptron (MLP), Gradient Boosting Machine (GBM), and Meta-Ensemble model. To account for the modest class imbalance in the training set (recurrence vs. non-recurrence ratio of approximately 1:2.1), model performance was comprehensively evaluated using balanced metrics, including the F1-score and the precision-recall area under the curve (PR-AUC). Hyperparameter tuning for each model was performed using 5-fold cross-validation on the training set to optimize the AUC. Model predictive performance was evaluated using receiver operating characteristic (ROC) curves, with 1,000 bootstrap iterations performed in the validation set to calculate the mean AUC and its 95% confidence interval (CI) for each model. The optimal classification threshold for each model was determined by maximizing the Youden index on the training set and subsequently applied to the validation set to calculate threshold-dependent metrics (accuracy, specificity, sensitivity, precision, recall, PPV, NPV, and F1-score). Model calibration was assessed by generating calibration curves on the validation set, and overall calibration performance was quantified using the Brier score. Additionally, a Meta-Ensemble model was constructed using a soft-voting strategy. Specifically, it integrated the top three performing base learners based on their AUC values in the training set. Unlike traditional stacking approaches that employ a meta-learner to learn how to best combine the predictions of base models, our soft-voting strategy directly averaged the predicted probabilities from the three selected base models to generate the final prediction. We retained the term ‘Meta-Ensemble’ to emphasize its ensemble nature, but it is technically a soft-voting classifier rather than a stacking model.

### 2.6 Model interpretability

Model interpretability was assessed using the SHAP (SHapley Additive exPlanations) method. SHAP values quantify the contribution of each feature to the model’s prediction: larger values indicate greater influence of the feature on prediction and higher importance in the model; conversely, smaller values indicate lesser influence. Furthermore, SHAP values can be positive or negative. Positive values indicate that the feature has a positive impact on the prediction, increasing the predicted value; negative values mean that the feature has a negative impact, decreasing the predicted value.

### 2.7 Statistical analysis

Data were processed using R (version 4.5.3). To ensure reproducibility, a fixed random seed [set.seed(42)] was set for all stochastic processes, including data splitting, cross-validation, and bootstrapping. Model training and hyperparameter tuning were implemented using the caret package. The base learners were built using the glm (for LR), xgboost, kernlab (for SVM), nnet (for MLP), and gbm packages. Hyperparameter grids were automatically searched by the caret package using 5-fold cross-validation (with tuneLength set to 3 or 4) to optimize the AUC. The Meta-Ensemble model was constructed by averaging the predicted probabilities of the top three base learners. Model evaluation (ROC, PR-AUC, calibration) was performed using the pROC and custom bootstrap scripts. Continuous variables were expressed as mean ± standard deviation (SD) for normally distributed data according to the Shapiro–Wilk normality test, with independent samples *t*-test for between-group comparisons; non-normally distributed data were expressed as median (interquartile range) [M (P_25_, P_75_)], with Mann–Whitney *U* test for between-group comparisons. Categorical variables were expressed as frequency and percentage [*n* (%)], with chi-square test or Fisher’s exact test for between-group comparisons based on expected frequencies. To further screen key variables, LASSO regression and the Boruta algorithm were used for dual feature selection. Based on the screened features, multiple machine learning models were constructed and trained in R (version 4.5.3) and evaluated. Meanwhile, the SHAP method was used to analyze feature importance of the optimal model and visualize the contribution of each feature to prediction. All statistical tests were two-sided, with *p* < 0.05 considered statistically significant.

## 3 Results

### 3.1 Characteristics of the study cohort

A total of 412 patients were enrolled and randomly divided into training and validation sets at a 7:3 ratio. The baseline characteristics of the training set are shown in Table 1.

**Table 1:** Baseline characteristics of the training set.

Variable	Non-recurrent (*n* = 196)	Recurrent (*n* = 93)	Test statistic	*p*-value
Age	58.92 ± 6.18	59.48 ± 6.66	*t* = −0.702	0.483
Residence			χ^2^ = 0.042	0.837
Rural	51 (26.0%)	26 (28.0%)		
Urban	145 (74.0%)	67 (72.0%)		
Education	8 (7, 9)	8 (6, 10)	*z* = 0.773	0.439
BMI	27.54 ± 2.57	26.84 ± 2.60	*t* = 2.160	0.032
Hypertension			χ^2^ = 0.015	0.903
No	142 (72.4%)	66 (71.0%)		
Yes	54 (27.6%)	27 (29.0%)		
Hyperlipidemia			χ^2^ = 2.212	0.137
No	158 (80.6%)	67 (72.0%)		
Yes	38 (19.4%)	26 (28.0%)		
Hyperglycemia			χ^2^ = 0.432	0.511
No	152 (77.6%)	76 (81.7%)		
Yes	44 (22.4%)	17 (18.3%)		
Migraine			χ^2^ = 9.965	0.002
No	138 (70.4%)	47 (50.5%)		
Yes	58 (29.6%)	46 (49.5%)		
Cervical spondylosis			χ^2^ = 0.222	0.637
No	160 (81.6%)	73 (78.5%)		
Yes	36 (18.4%)	20 (21.5%)		
Menieres disease			χ^2^ = 2.835	0.092
No	158 (80.6%)	66 (71.0%)		
Yes	38 (19.4%)	27 (29.0%)		
Osteoporosis			χ^2^ = 10.732	0.001
No	143 (73.0%)	49 (52.7%)		
Yes	53 (27.0%)	44 (47.3%)		
Otolith position			χ^2^ = 3.237	0.357
Posterior SCC	83 (42.3%)	35 (37.6%)		
Horizontal SCC	49 (25.0%)	26 (28.0%)		
Anterior SCC	33 (16.8%)	22 (23.7%)		
Mixed type	31 (15.8%)	10 (10.8%)		
DHI			χ^2^ = 4.328	0.115
Mild	79 (40.3%)	31 (33.3%)		
Moderate	74 (37.8%)	47 (50.5%)		
Severe	43 (21.9%)	15 (16.1%)		
HADS-A			χ^2^ = 9.489	0.002
Normal	150 (76.5%)	54 (58.1%)		
Abnormal	46 (23.5%)	39 (41.9%)		
HADS-D			χ^2^ = 1.524	0.217
Normal	165 (84.2%)	72 (77.4%)		
Abnormal	31 (15.8%)	21 (22.6%)		
PSQI			χ^2^ = 2.835	0.092
Normal	158 (80.6%)	66 (71.0%)		
Abnormal	38 (19.4%)	27 (29.0%)		
VAS	5 (4, 6)	5 (4, 6)	*z* = 0.845	0.398
Calcium	2.63 (1.98, 3.24)	1.57 (1.22, 2.34)	*z* = 6.789	<0.001
Vitamin D	20.19 (18.31, 21.92)	17.31 (15.93, 18.84)	*z* = 7.055	<0.001
Estradiol	17.79 (15.92, 19.49)	14.06 (12.55, 16.09)	*z* = 7.289	<0.001

### 3.2 Machine learning feature selection

**Figure 1:**
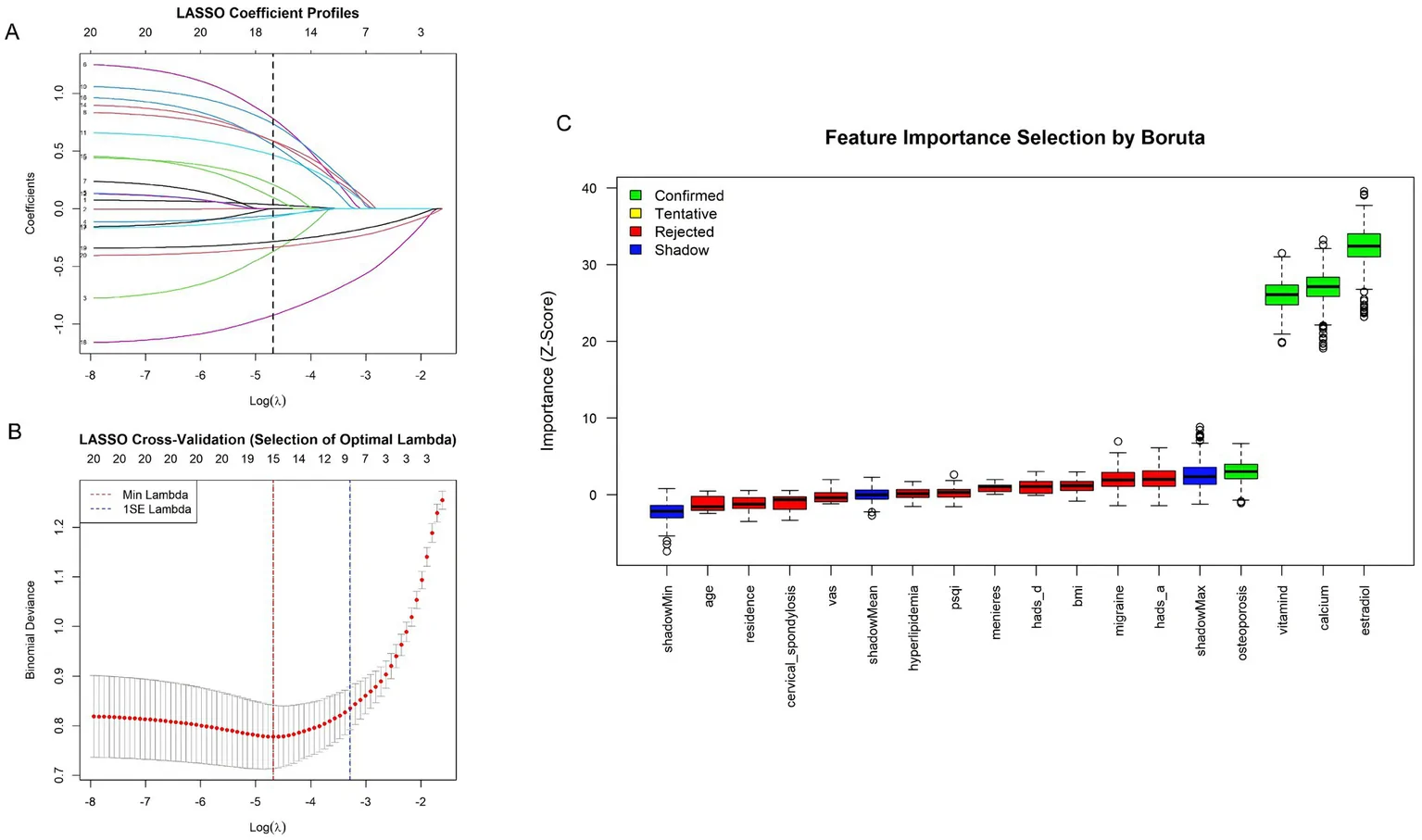
Feature selection using Boruta and LASSO regression. **(A)** LASSO coefficient profiles. **(B)** LASSO cross-validation error curve. **(C)** Boruta feature importance plot. VAS, Visual Analog Scale; PSQI, Pittsburgh Sleep Quality Index; HADS-D, Hospital Anxiety and Depression Scale–Depression subscale; BMI, Body Mass Index; HADS-A, Hospital Anxiety and Depression Scale–Anxiety subscale.

**Figure 2:**
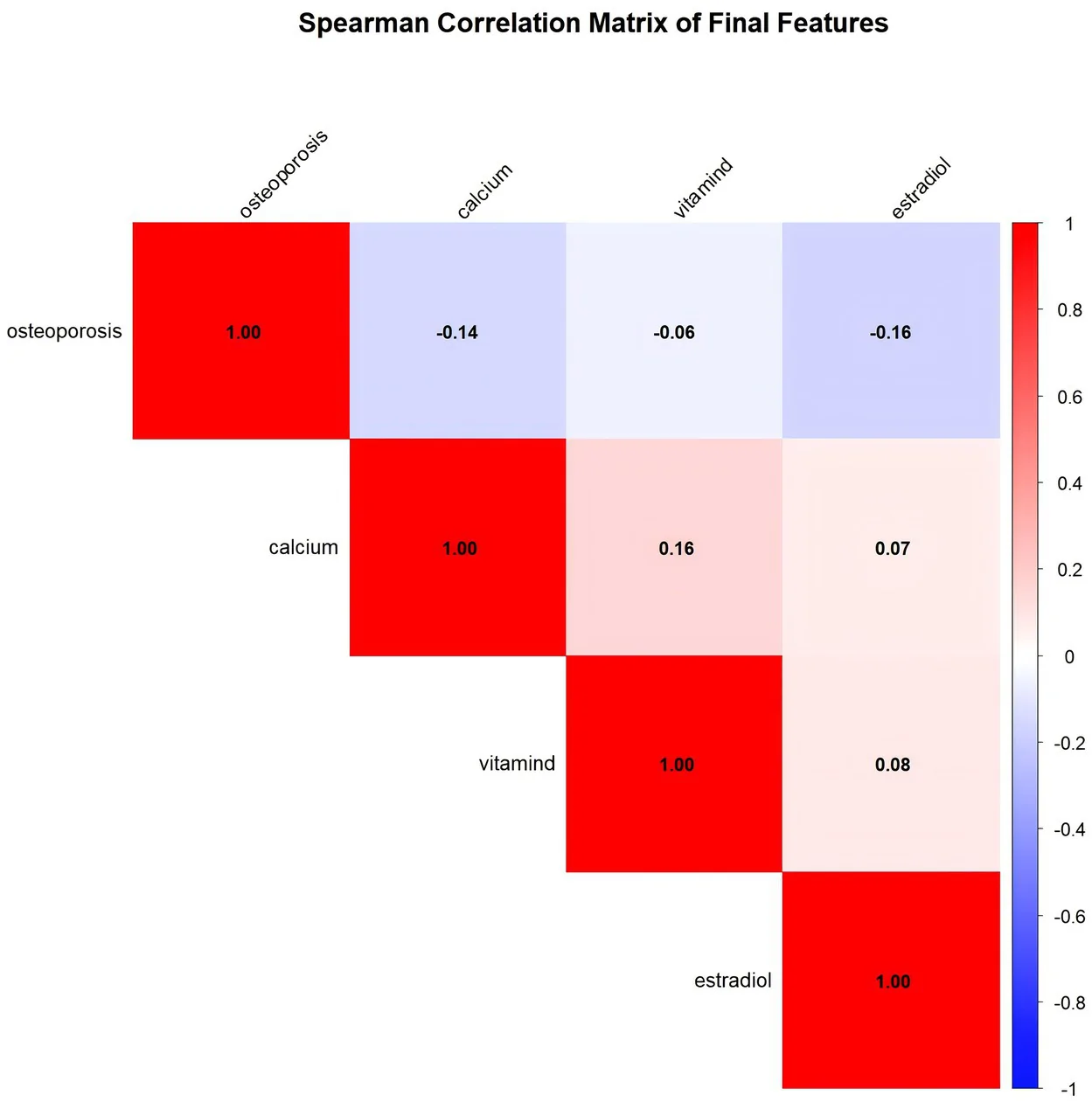
Spearman correlation analysis among osteoporosis, serum calcium, vitamin D, and estradiol levels.

LASSO regression and the Boruta algorithm were used for feature screening. LASSO regression screened 15 variables from 20 variables in the training set as potential predictors: age, residence, cervical spondylosis, VAS, hyperlipidemia, PSQI, Meniere’s disease, HADS-D, BMI, migraine, HADS-A, osteoporosis, vitamin D level, serum calcium level, and estradiol level. Further feature importance assessment through the Boruta algorithm ultimately identified four model-selected predictors: osteoporosis, serum calcium, vitamin D level, and estradiol level (Figures 1A–C). Spearman correlation analysis showed (Figure 2) that the correlation coefficients between any two predictive variables were low (|*r*| ≤ 0.16), indicating limited pairwise multicollinearity among the selected variables, suitable for joint inclusion in subsequent machine learning models for modeling analysis.

### 3.3 Model construction and validation

**Figure 3:**
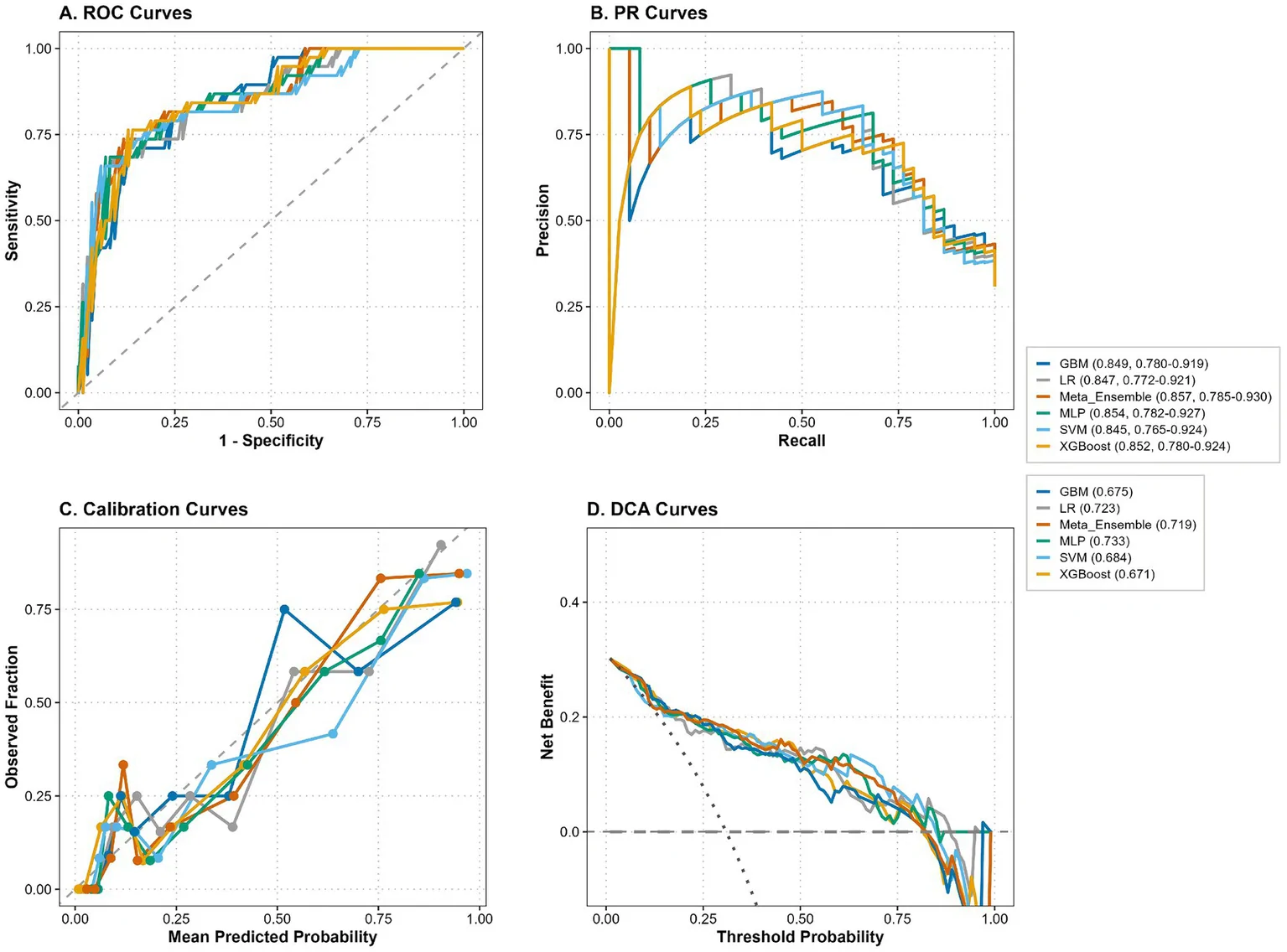
Performance analysis of six machine learning models. **(A)** ROC curves. **(B)** PR-AUC curves. **(C)** Calibration curves. **(D)** Clinical decision curves.

This study developed and evaluated six fundamental machine learning models (LR, XGBoost, SVM, MLP, GBM, and Meta-Ensemble). All models showed excellent discrimination on the test set (AUC > 0.84, Figure 3A), with the Meta-Ensemble model, constructed by averaging the predicted probabilities from the top three base learners (GBM, XGBoost, and SVM), achieving the highest AUC (0.857, 95% CI: 0.779–0.928). DeLong’s test (Supplementary Table 2) revealed no significant differences between any two models (all *p* > 0.05), indicating comparable discrimination; however, the Meta-Ensemble maintained the highest absolute AUC. For threshold-dependent metrics (Table 2), the Meta-Ensemble achieved a balanced performance (sensitivity = 0.790, specificity = 0.800, F1 = 0.706). While XGBoost yielded identical specificity, accuracy, and F1-score, along with the highest Youden index (0.634), and MLP achieved the highest precision-recall area under the curve (PR-AUC) (0.733, Figure 3B), the Meta-Ensemble provided a more harmonized overall performance. The calibration curve (Figure 3C) showed good agreement between predicted and actual probabilities. Decision curve analysis (Figure 3D) confirmed its clinical utility across a wide range of threshold probabilities. Given its leading discrimination, clinical benefit, and the ensemble’s theoretical advantage in mitigating overfitting, the Meta-Ensemble was selected as the preferred model.

**Table 2:** Comparison of threshold-based performance metrics across six machine learning modes.

Model	AUC (95% CI)	PR-AUC	Specificity	Sensitivity	PPV	NPV	Accuracy	F1-score	Youden_J	Threshold
LR	0.847 (0.767–0.917)	0.723	0.671	0.816	0.525	0.891	0.715	0.639	0.590	0.259
XGBoost	0.852 (0.769–0.923)	0.671	0.800	0.790	0.638	0.895	0.797	0.706	0.634	0.337
SVM	0.845 (0.761–0.923)	0.684	0.718	0.816	0.564	0.897	0.748	0.667	0.599	0.194
MLP	0.854 (0.776–0.922)	0.733	0.765	0.790	0.600	0.890	0.772	0.682	0.614	0.311
GBM	0.849 (0.771–0.916)	0.675	0.718	0.816	0.564	0.897	0.748	0.667	0.569	0.245
Meta-Ensemble	0.857 (0.779–0.928)	0.719	0.800	0.790	0.638	0.895	0.797	0.706	0.619	0.326

LR, Logistic Regression; XGBoost, eXtreme Gradient Boosting; SVM, Support Vector Machine; MLP, Multilayer Perceptron; GBM, Gradient Boosting Machine; AUC, Area Under the Curve; CI, Confidence Interval; PR-AUC, Precision-Recall AUC; PPV, Positive Predictive Value; NPV, Negative Predictive Value.

### 3.4 Evaluation of the optimal model

**Figure 4:**
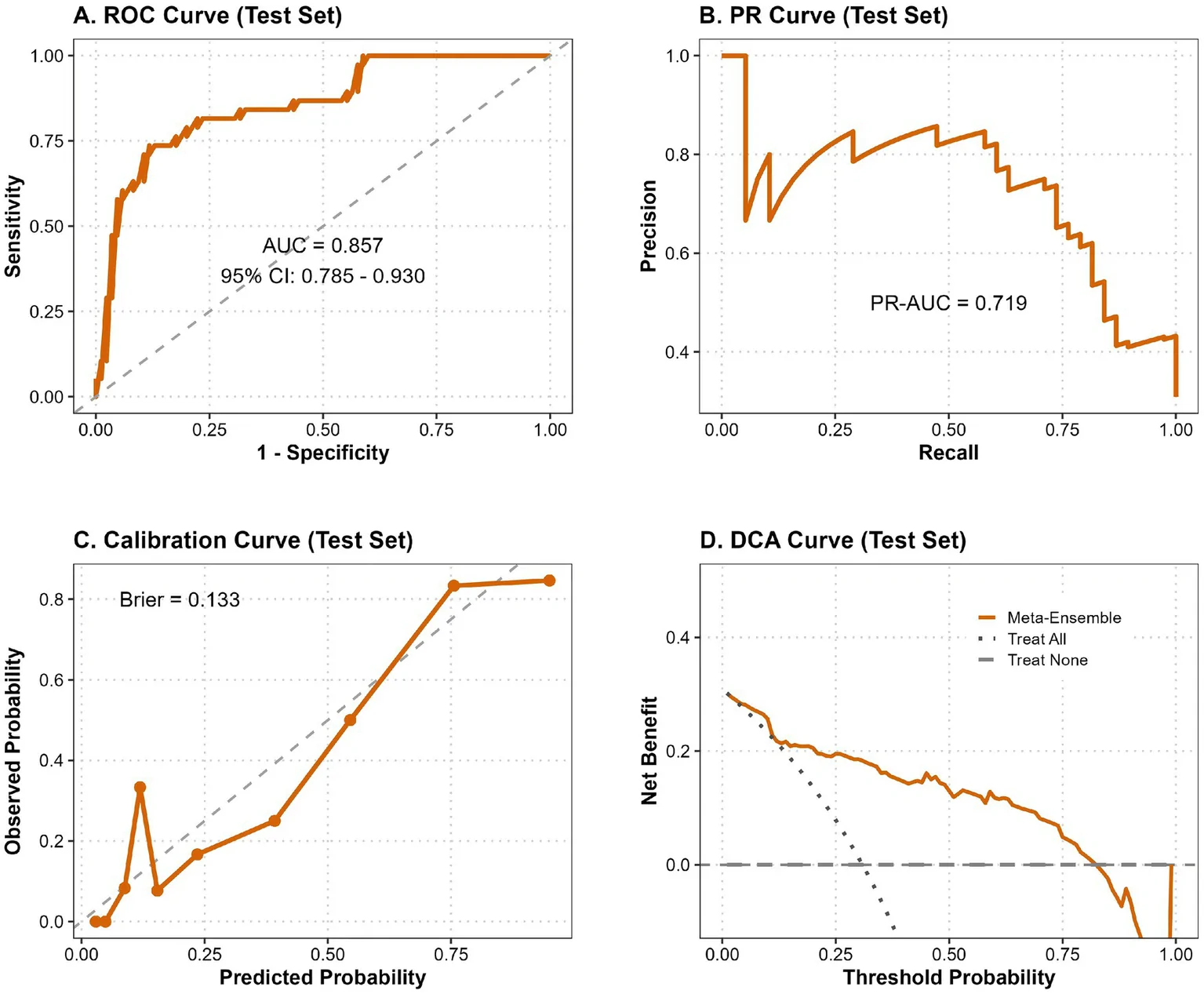
Performance analysis of the Meta-Ensemble model **(A)** ROC curve **(B)** PR-AUC curve **(C)** Calibration curve **(D)** Clinical decision curve.

Figure 4 presents the performance analysis of the Meta-Ensemble model on the test set, including ROC, PR, calibration, and decision curve analyses. The ROC curve (Figure 4A) shows an AUC of 0.857 (95% CI: 0.785–0.930), with the curve rising steeply from the origin and approaching the upper left corner, indicating strong discriminative ability. The PR curve (Figure 4B) has a PR-AUC of 0.719, where precision remains relatively high at lower recall values and gradually decreases as recall increases, reflecting balanced precision-recall trade-offs. The calibration curve (Figure 4C) displays a Brier score of 0.133, closely following the diagonal reference line, suggesting good agreement between predicted probabilities and observed outcomes. The decision curve analysis (Figure 4D) reveals that the Meta-Ensemble model yields a positive net benefit across a wide range of threshold probabilities (approximately 0.05–0.85), outperforming the “Treat None” strategy and approaching the “Treat All” strategy in specific intervals, demonstrating its clinical utility.

### 3.5 Interpretation of the optimal model

**Figure 5:**
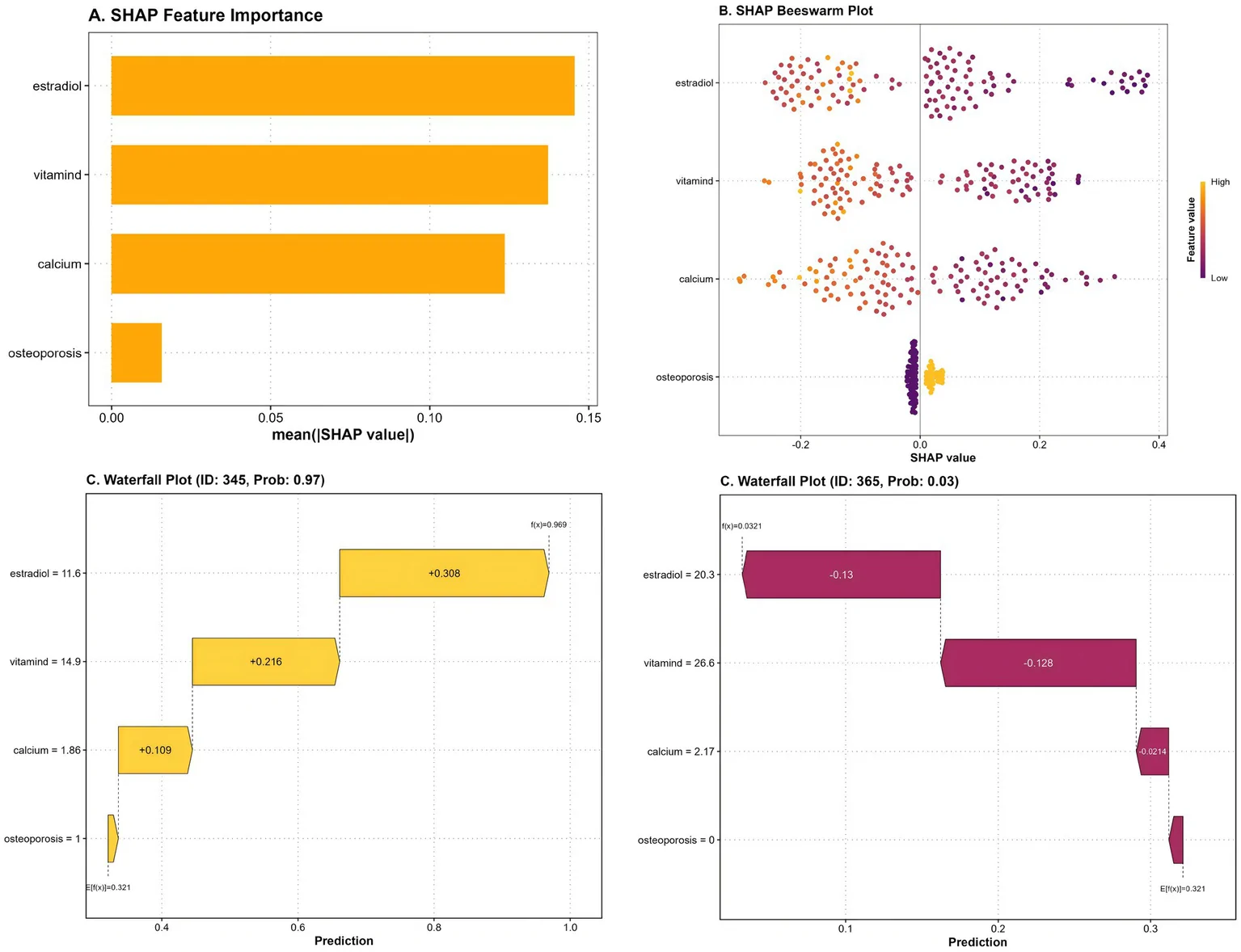
SHAP interpretability analysis for the Meta-Ensemble model. **(A)** Feature importance ranking. **(B)** Beeswarm plot. **(C)** Waterfall plot for a high-risk individual (Sample 345). **(D)** Waterfall plot for a low-risk individual (Sample 365).

This study used the SHAP method for interpretability analysis of the BPPV recurrence prediction model (Figures 5A–D). The SHAP feature importance bar plot demonstrates that estradiol is the most critical feature influencing model prediction (with an average absolute SHAP value of approximately 0.14), followed by vitamin D (approximately 0.12) and calcium (approximately 0.10), while osteoporosis contributes the least to prediction (approximately 0.02). The beeswarm plot further revealed that low vitamin D, low serum calcium, low estradiol, and concomitant osteoporosis all corresponded to positive SHAP values, pushing the model output toward high recurrence risk; conversely, higher levels of these indicators were associated with negative SHAP values, indicating an association with lower predicted recurrence risk in the model (Figure 5B). At the individual level, SHAP waterfall plots visually demonstrated feature-driven risk differences. Taking Sample 345 (predicted recurrence probability 97%) as an example, low estradiol (+0.308), low vitamin D (+0.216), low serum calcium (+0.109), and osteoporosis (+0.015) ultimately raised the predicted probability to 0.97 (Figure 5C); while Sample 365 (predicted recurrence probability 3%) had relatively high estradiol (−0.130), vitamin D (−0.128), serum calcium (−0.021) and absence of osteoporosis (−0.009), resulting in a predicted probability of only 0.03 (Figure 5D).

**Figure 6:**
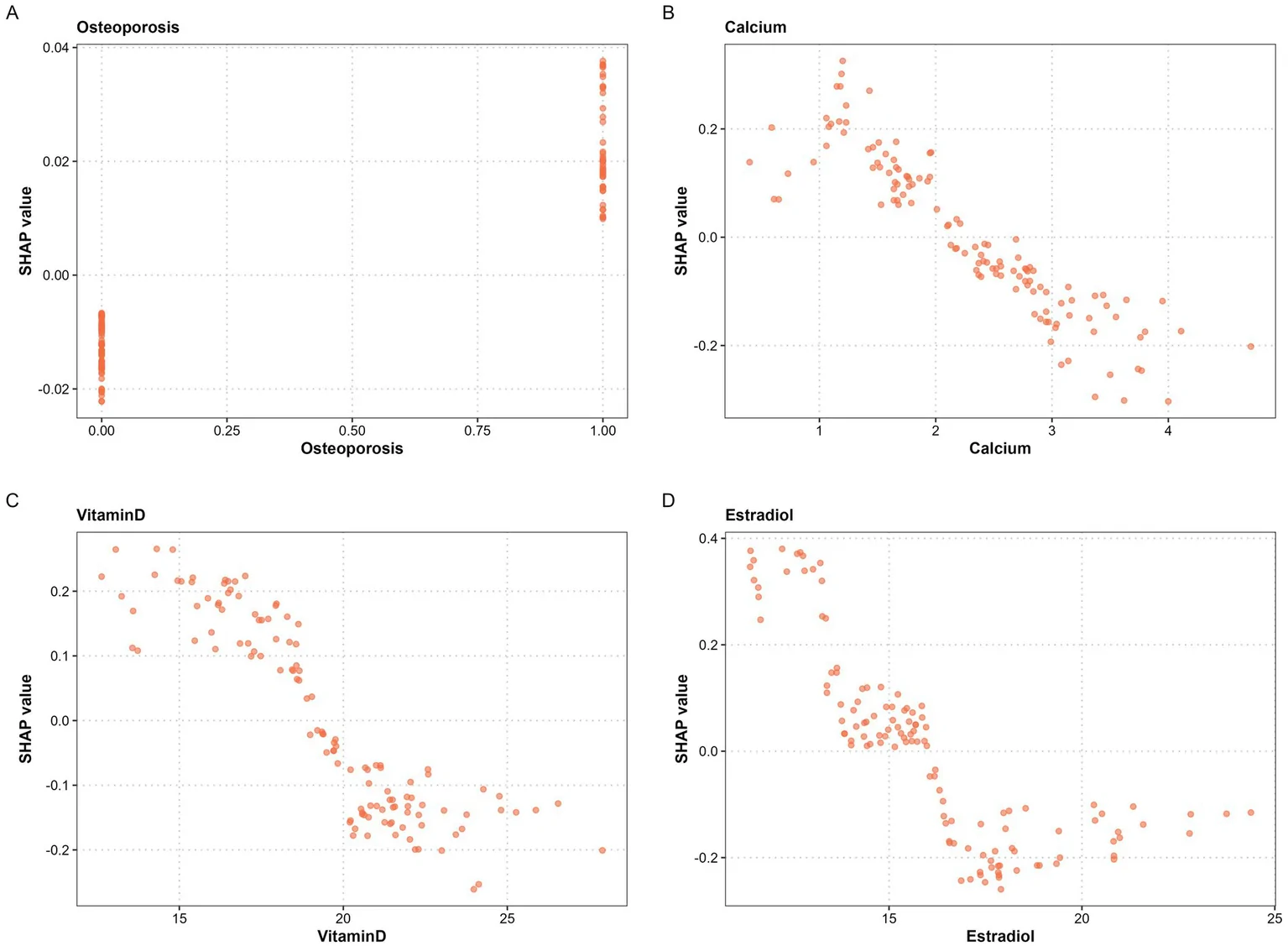
Relationship between the four feature values and SHAP values. **(A)** Osteoporosis versus SHAP value. **(B)** Calcium versus SHAP value. **(C)** Vitamin D versus SHAP value. **(D)** Estradiol versus SHAP value.

The relationship between the four model-selected predictors and SHAP values is shown in Figure 6. SHAP values >0 indicate pushing the model prediction toward high BPPV recurrence risk, <0 indicate low risk direction.

To serve as a model demonstration tool and a potential clinical decision-support aid, this study developed a web-based calculator based on the final Meta-Ensemble model (which exclusively utilizes the four selected features). Users input four variables: osteoporosis status (Yes/No), serum calcium (mmol/L), vitamin D (ng/mL), and estradiol (pg/mL). The calculator applies the same preprocessing steps (mean imputation and standardization) used during model training and averages the predicted probabilities from the XGBoost, GBM, and SVM base learners to output a recurrence probability. Risk stratification is determined using pre-specified thresholds: probability ≥0.8 indicates “High Risk” (recommending close follow-up and active intervention), 0.5–0.79 indicates “Moderate Risk” (recommending regular follow-up), and <0.5 indicates “Low Risk” (recommending routine check-up). The figure illustrates a prediction example for a high-risk individual (Sample ID: 345): this patient has concomitant osteoporosis, with low vitamin D at 14.95 ng/mL, serum calcium at 1.86 mmol/L, and estradiol at 11.61 pg./mL; the model outputs a recurrence probability of 89.80%, and the system indicates “High Risk” with a recommendation for close follow-up and active intervention. The Uniform Resource Locator^1^.

## 4 Discussion

**Figure 7:**
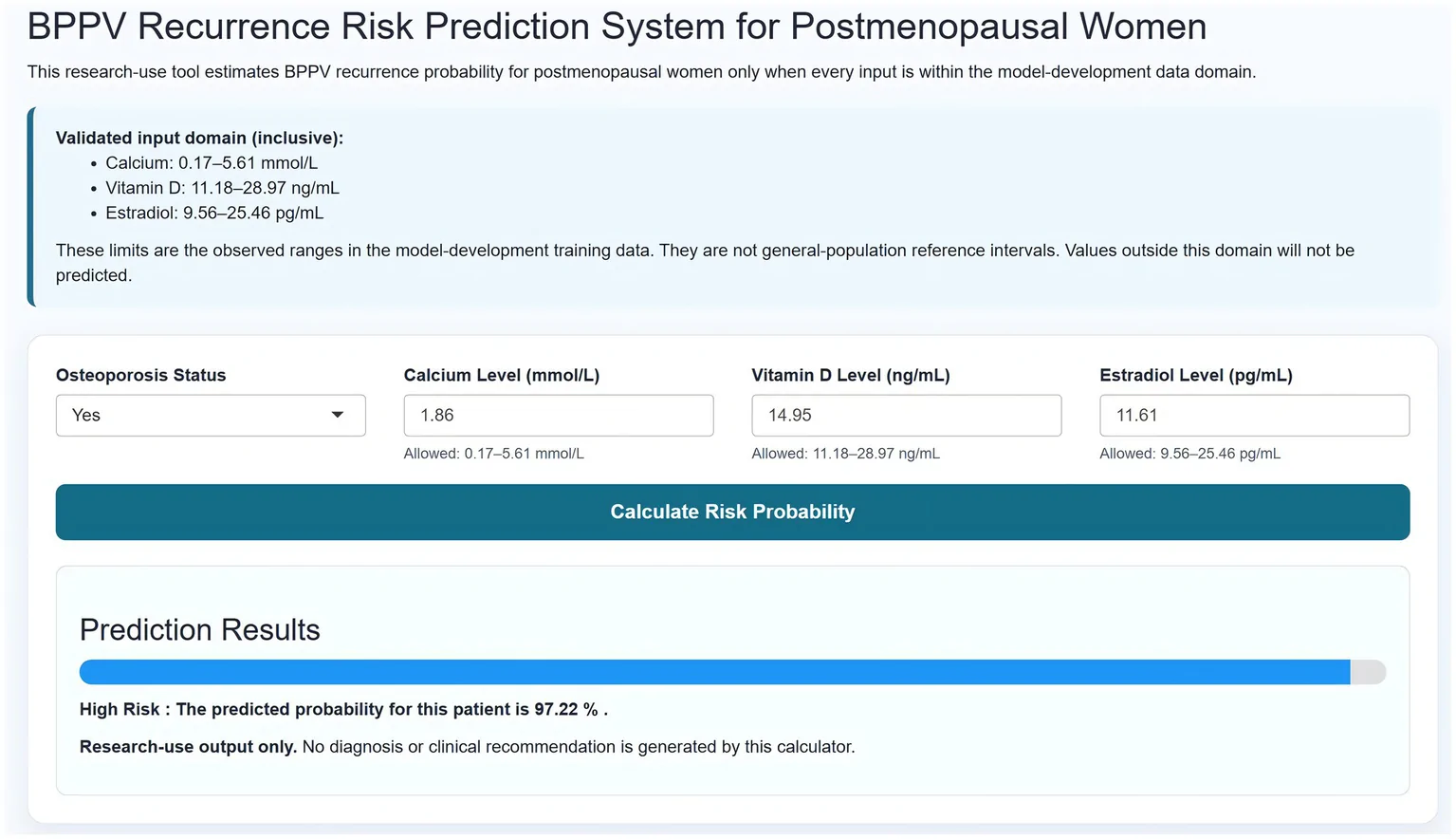
Web-based BPPV recurrence risk prediction system for postmenopausal women based on the Meta-Ensemble model.

The pathogenesis of BPPV is related to otoconia detachment and semicircular canal endolymph fluid dynamics disturbance, which subsequently disrupts vestibular balance function and induces vertigo (11). Postmenopausal women are prone to abnormal bone metabolism and otoconial organ degeneration due to decreased estrogen levels, making them a high-risk population for BPPV recurrence (Figure 7).

Our use of the Meta-Ensemble model and LASSO + Boruta feature selection aligns with state-of-the-art approaches in clinical prediction, as demonstrated in recent studies using machine learning for disease risk assessment (18). The integration of SHAP analysis further enhances model interpretability, a critical requirement for clinical adoption (19). This study systematically compared the predictive performance of six machine learning models for BPPV recurrence in postmenopausal women. The core findings include: (1) LASSO and Boruta dual algorithms jointly screened four model-selected predictors: osteoporosis, serum calcium, 25(OH)D, and estradiol; (2) With an AUC of 0.857, the Meta-Ensemble model demonstrated the highest discriminative ability. We acknowledge that the AUC of the Meta-Ensemble is almost identical to that of XGBoost (0.852), and DeLong’s test indicated no statistically significant difference between them. Therefore, we do not claim that the Meta-Ensemble is definitively superior to XGBoost in terms of discrimination. However, according to the threshold-based metrics (Table 2), while XGBoost achieved the highest Youden’s index (0.634), the Meta-Ensemble provided a more harmonized overall performance, balancing high sensitivity (0.790) and specificity (0.800) without sacrificing overall accuracy (0.797) or F1-score (0.706). Ultimately, rather than claiming superiority, we adopted the Meta-Ensemble as the preferred model owing to its theoretical robustness against overfitting and its capacity to integrate diverse algorithmic perspectives, yielding more stable generalization performance; (3) SHAP analysis confirmed that low vitamin D, low serum calcium, low estradiol, and concomitant osteoporosis are key factors driving elevated recurrence risk, with weak correlations among the four (Spearman *r* ≤ 0.16). The four model-selected predictors identified in this study are highly consistent with previous literature. Yamanaka et al. (2) found that the BPPV recurrence rate in patients with osteoporosis (56.3%) was significantly higher than in those with normal bone density (16.1%); Yang et al. (20) confirmed that estradiol deficiency increases recurrence risk by downregulating otoconial protein otoconin-90 expression; Jeong et al. (9) and Han et al. (10) respectively reported associations between low vitamin D levels and BPPV occurrence and recurrence; Pan et al. (8) similarly incorporated 25(OH)D, calcium, and estradiol in their Logistic regression nomogram. Compared with previous reports evaluating BPPV recurrence prediction using traditional statistical methods or specific biomarkers (21–23), the present model showed a relatively high AUC. However, direct superiority cannot be concluded without external benchmark validation, as these studies differ in population characteristics, outcome definitions, predictor sets, and validation methods. Nonetheless, the performance of our model may benefit from our study focusing on postmenopausal women as the research subjects, reducing heterogeneity in gender and hormone levels, combined with LASSO + Boruta dual algorithm for variable screening that accounts for both linear and nonlinear associations, and ultimately employing meta-ensemble strategy to effectively integrate multiple base learners, reducing overfitting risk.

This study differs somewhat from Pan et al. (8) in that their study identified migraine as an independent risk factor (OR = 2.208), but migraine did not pass Boruta screening in our study. The possible reasons are: the age of our study population was relatively high, migraine prevalence decreases with increasing age, while the dominant role of metabolic factors is relatively enhanced; in multivariable competition, the predictive contribution of migraine was diluted by the four metabolic indicators, suggesting the hypothesis that it may influence recurrence through indirect pathways (such as affecting sleep or mood) rather than directly acting on otoconial metabolic pathways, which requires further investigation.

Machine learning has emerged as a transformative tool in medicine, enabling the development of accurate and interpretable prediction models (24). Based on the Meta-Ensemble model, this study developed a web-based simplified calculator for BPPV recurrence risk in postmenopausal women. By inputting osteoporosis status (yes/no), serum calcium, vitamin D, and estradiol values, the recurrence probability can be automatically output. This tool requires no programming knowledge and serves as a potential clinical decision-support aid for clinicians, though further external validation and usability testing are required before widespread clinical implementation.

However, this study has the following limitations: First, the retrospective design may have selection bias, and the history of vitamin D, calcium supplement, or anti-osteoporosis treatment in some patients was difficult to fully adjust for. Second, although the sample size (412 cases) met modeling requirements, it is still relatively small given the complexity of ensemble learning, with potential overfitting risk. Third, the data came from only three hospitals in Sichuan Province, lacking external validation; the generalizability of the model in northern China or other ethnic populations remains unclear. Future research should prioritize prospective, multicenter, large-sample external validation, extending follow-up to 1–2 years to confirm the model’s generalizability and long-term predictive performance. Future randomized controlled trials could be designed to evaluate the efficacy of targeted clinical assessments and risk-informed interventions (e.g., prospective trials of metabolic or hormonal management) for high-risk patients, verifying whether such strategies based on the model can truly reduce BPPV recurrence rates. This model provides a quantifiable assessment tool for recurrence risk management of BPPV in postmenopausal women and has certain potential for clinical application.

## Data Availability

The raw data supporting the conclusions of this article will be made available by the authors, without undue reservation.

## Footnotes

1
https://019f7d58-a152-5560-c18b-acf6e1b6b503.share.connect.posit.cloud/
