# Supplementary material for: Prediction of benign paroxysmal positional vertigo recurrence in postmenopausal women: a machine learning-based clinical study
Source: Front Neurol. 2026 Aug 25;17:1890770. doi: 10.3389/fneur.2026.1890770 (PMC13549858; doi:10.3389/fneur.2026.1890770)
Supplement: Supplementary file 1 [file Data_Sheet_1.ZIP › supplementary materials/DeLong Test.docx]

Supplementary Table2 Pairwise comparison of AUCs among six machine learning models using DeLong’s test

| Model | LR | XGBoost | SVM | MLP | GBM | Meta_Ensemble |
| --- | --- | --- | --- | --- | --- | --- |
| LR | 1 | 0.7709 | 0.8365 | 0.171 | 0.8854 | 0.3895 |
| XGBoost | 0.7709 | 1 | 0.6973 | 0.9119 | 0.828 | 0.6309 |
| SVM | 0.8365 | 0.6973 | 1 | 0.3978 | 0.8095 | 0.2996 |
| MLP | 0.171 | 0.9119 | 0.3978 | 1 | 0.7634 | 0.8297 |
| GBM | 0.8854 | 0.828 | 0.8095 | 0.7634 | 1 | 0.4904 |
| Meta_Ensemble | 0.3895 | 0.6309 | 0.2996 | 0.8297 | 0.4904 | 1 |

LR, Logistic Regression; XGBoost, eXtreme Gradient Boosting; SVM, Support Vector Machine; MLP, Multilayer Perceptron; GBM, Gradient Boosting Machine. The values in the table represent the P-values from DeLong’s test comparing the area under the receiver operating characteristic curve (AUC) between each pair of models on the validation set. P-value < 0.05 was considered statistically significant.
