# Supplementary material for: Prediction of benign paroxysmal positional vertigo recurrence in postmenopausal women: a machine learning-based clinical study
Source: Front Neurol. 2026 Aug 25;17:1890770. doi: 10.3389/fneur.2026.1890770 (PMC13549858; doi:10.3389/fneur.2026.1890770)
Supplement: Supplementary file 1 [file Data_Sheet_1.ZIP › supplementary materials/Machine learning——R code.docx]

\# ==================== 0. 环境准备 ====================

cat("正在检查并修复R包环境...\n")

options(repos = c(CRAN = "https://mirrors.tuna.tsinghua.edu.cn/CRAN/"))

required_packages <- c("readxl", "dplyr", "caret", "pROC", "xgboost", "e1071",

"nnet", "gbm", "ggplot2", "patchwork", "rlang", "vctrs", "scales",

"RColorBrewer", "shapviz")

for (pkg in required_packages) {

if (!requireNamespace(pkg, quietly = TRUE)) {

cat(sprintf(" -> 正在安装缺失的包: %s\n", pkg))

install.packages(pkg, dependencies = TRUE)

}

}

suppressPackageStartupMessages({

library(readxl)

library(dplyr)

library(caret)

library(pROC)

library(xgboost)

library(e1071)

library(nnet)

library(gbm)

library(ggplot2)

library(patchwork)

library(scales)

library(RColorBrewer)

library(shapviz)

})

\# ==================== 1. 数据加载与清洗 ====================

cat("正在加载数据...\n")

\# 修复1: 统一变量名为 df_train 和 df_test

df_train <- read_excel("C:/BPPV修改/修改/训练集.xlsx")

df_test <- read_excel("C:/BPPV修改/修改/测试集.xlsx")

colnames(df_train) <- tolower(trimws(colnames(df_train)))

colnames(df_test) <- tolower(trimws(colnames(df_test)))

target_col <- "type"

id_col <- "id"

features <- setdiff(colnames(df_train), c(target_col, id_col))

cat("建模使用的特征变量:", paste(features, collapse=", "), "\n")

X_train <- df_train[, features, drop = FALSE]

y_train <- as.numeric(df_train[[target_col]])

X_test <- df_test[, features, drop = FALSE]

y_test <- as.numeric(df_test[[target_col]])

for(f in features) {

X_train[[f]] <- as.numeric(X_train[[f]])

X_test[[f]] <- as.numeric(X_test[[f]])

}

train_valid <- !is.na(y_train); X_train <- X_train[train_valid, , drop = FALSE]; y_train <- y_train[train_valid]

test_valid <- !is.na(y_test); X_test <- X_test[test_valid, , drop = FALSE]; y_test <- y_test[test_valid]

\# ==================== 2. 数据预处理 ====================

cat("正在进行数据预处理 (均值填补)...\n")

train_means <- sapply(X_train, mean, na.rm = TRUE)

for(f in features) {

X_train[[f]][is.na(X_train[[f]])] <- train_means[f]

X_test[[f]][is.na(X_test[[f]])]	<- train_means[f]

}

train_df <- X_train

train_df$type <- factor(ifelse(y_train == 1, "Yes", "No"), levels = c("No", "Yes"))

ctrl <- trainControl(method = "cv", number = 5, classProbs = TRUE,

summaryFunction = twoClassSummary, savePredictions = "final")

preprocess_methods <- c("center", "scale")

\# ==================== 3. 模型训练 (5个基础模型) ====================

set.seed(42); cat("正在训练 LR...\n")

model_lr <- train(type ~ ., data = train_df, method = "glm", family = "binomial",

trControl = ctrl, preProcess = preprocess_methods, metric = "ROC")

set.seed(42); cat("正在训练 XGBoost...\n")

model_xgb <- train(type ~ ., data = train_df, method = "xgbTree",

trControl = ctrl, preProcess = preprocess_methods, metric = "ROC",

tuneLength = 4, verbosity = 0)

set.seed(42); cat("正在训练 SVM...\n")

model_svm <- train(type ~ ., data = train_df, method = "svmRadial",

trControl = ctrl, preProcess = preprocess_methods, metric = "ROC", tuneLength = 4)

set.seed(42); cat("正在训练 MLP...\n")

model_mlp <- train(type ~ ., data = train_df, method = "nnet",

trControl = ctrl, preProcess = preprocess_methods, metric = "ROC",

tuneLength = 3, trace = FALSE, maxit = 500)

set.seed(42); cat("正在训练 GBM...\n")

model_gbm <- train(type ~ ., data = train_df, method = "gbm",

trControl = ctrl, preProcess = preprocess_methods, metric = "ROC",

tuneLength = 3, verbose = FALSE)

\# ==================== 4. 构建 Meta-Ensemble + 组成模型检查 ====================

cat("\n============================================================\n")

cat("	Meta-Ensemble 组成模型检查报告\n")

cat("============================================================\n\n")

cv_aucs <- c(LR = max(model_lr$results$ROC), XGBoost = max(model_xgb$results$ROC),

SVM = max(model_svm$results$ROC), MLP = max(model_mlp$results$ROC),

GBM = max(model_gbm$results$ROC))

cat("【第1步】5个基础模型的交叉验证AUC排名：\n")

cv_auc_df <- data.frame(Rank = 1:5, Model = names(sort(cv_aucs, decreasing = TRUE)), CV_AUC = round(sort(cv_aucs, decreasing = TRUE), 4))

print(cv_auc_df, row.names = FALSE)

top3_models <- names(sort(cv_aucs, decreasing = TRUE)[1:3])

cat("\n【第2步】实际选入 Meta-Ensemble 的 Top 3 模型：\n")

for(i in seq_along(top3_models)) {

cat(sprintf(" Top %d: %-12s (CV AUC = %.4f)\n", i, top3_models[i], cv_aucs[top3_models[i]]))

}

cat("\n【第3步】与论文预期对比：\n")

paper_top3 <- c("XGBoost", "LR", "SVM")

cat(sprintf(" 论文预期 Top 3: %s\n", paste(paper_top3, collapse = ", ")))

cat(sprintf(" 代码实际 Top 3: %s\n", paste(top3_models, collapse = ", ")))

if(identical(sort(top3_models), sort(paper_top3))) {

cat("\n ✅ 结论：完全一致！Meta-Ensemble 由 XGBoost + LR + SVM 三个模型软投票组成。\n")

} else {

cat("\n ⚠️ 结论：存在差异！代码使用训练集CV_AUC排序，论文使用验证集AUC排序。\n")

cat("	如需与论文完全一致，可强制指定: top3_models <- c('XGBoost', 'LR', 'SVM')\n")

}

cat("============================================================\n\n")

base_models <- list(LR=model_lr, XGBoost=model_xgb, SVM=model_svm, MLP=model_mlp, GBM=model_gbm)

model_names <- c(names(base_models), "Meta_Ensemble")

get_probs <- function(model, newdata) as.numeric(unlist(predict(model, newdata, type="prob")$Yes))

\# ==================== 保存模型供网页使用 ====================

cat("\n正在保存模型文件...\n")

saveRDS(model_xgb, file = "C:/BPPV修改/修改/model_xgb.rds")

saveRDS(model_gbm, file = "C:/BPPV修改/修改/model_gbm.rds")

saveRDS(model_svm, file = "C:/BPPV修改/修改/model_svm.rds")

cat("模型文件已保存至 C:/BPPV修改/修改/\n")

cat(" - model_xgb.rds\n")

cat(" - model_gbm.rds\n")

cat(" - model_svm.rds\n")

\# ==================== 5. 锁定训练集阈值 ====================

cat("\n----------------------------------------------------\n")

cat("【关键步骤】正在基于训练集计算并锁定最佳截断值...\n")

cat("----------------------------------------------------\n")

probs_train_all <- data.frame(sapply(names(base_models), function(m) get_probs(base_models[[m]], X_train)))

probs_train_all$Meta_Ensemble <- rowMeans(probs_train_all[, top3_models])

train_thresholds <- list()

for(m in model_names) {

roc_train <- pROC::roc(y_train, probs_train_all[[m]], quiet=TRUE, levels=c(0,1), direction="<")

best_thresh <- as.numeric(pROC::coords(roc_train, "best", ret="threshold", best.method="youden")[1])

train_thresholds[[m]] <- best_thresh

cat(sprintf(" - 模型 %-15s 的训练集最佳阈值: %.4f\n", m, best_thresh))

}

\# ==================== 6. 核心评估函数 ====================

calc_auc_manual <- function(y, p) {

pos <- p[y==1]; neg <- p[y==0]

if(length(pos)==0 || length(neg)==0) return(0.5)

r <- rank(c(pos, neg))

(sum(r[1:length(pos)]) - length(pos)*(length(pos)+1)/2) / (length(pos)*length(neg))

}

calc_metrics <- function(y, p, thresh, model, dataset) {

y <- as.numeric(unlist(y)); p <- as.numeric(unlist(p))

valid_idx <- !is.na(p) & !is.na(y)

y <- y[valid_idx]; p <- p[valid_idx]

y_pred <- ifelse(p >= thresh, 1, 0)

tp <- sum(y==1 & y_pred==1); fp <- sum(y==0 & y_pred==1)

tn <- sum(y==0 & y_pred==0); fn <- sum(y==1 & y_pred==0)

auc <- calc_auc_manual(y, p)

set.seed(42)

boot_aucs <- replicate(1000, { idx <- sample(length(y), replace=TRUE); if(length(unique(y[idx]))<2) return(NA); calc_auc_manual(y[idx], p[idx]) })

boot_aucs <- boot_aucs[!is.na(boot_aucs)]

auc_low <- quantile(boot_aucs, 0.025); auc_high <- quantile(boot_aucs, 0.975)

ord <- order(p, decreasing=TRUE)

tp_cum <- cumsum(y[ord]==1); fp_cum <- cumsum(y[ord]==0)

prec <- tp_cum/(tp_cum+fp_cum); rec <- tp_cum/sum(y==1)

pr_auc <- sum(diff(c(0,rec)) * c(1, head(prec,-1)))

\# 修正：使用coords函数直接获取最佳阈值对应的敏感性和特异性

roc_obj <- pROC::roc(y, p, quiet=TRUE, levels=c(0,1), direction="<")

coords_result <- pROC::coords(roc_obj, "best", ret=c("sensitivity", "specificity"), best.method="youden")

sensitivity <- coords_result$sensitivity

specificity <- coords_result$specificity

youden_j <- sensitivity + specificity - 1

data.frame(

Dataset=dataset, Model=model,

AUC=sprintf("%.3f (%.3f-%.3f)", auc, auc_low, auc_high),

PR_AUC=sprintf("%.3f", pr_auc), Threshold=round(thresh, 4),

Sensitivity=round(ifelse(tp+fn>0, tp/(tp+fn), 0), 4),

Specificity=round(ifelse(tn+fp>0, tn/(tn+fp), 0), 4),

PPV=round(ifelse(tp+fp>0, tp/(tp+fp), 0), 4),

NPV=round(ifelse(tn+fn>0, tn/(tn+fn), 0), 4),

Accuracy=round((tp+tn)/length(y), 4),

F1=round(ifelse(2*tp+fp+fn>0, 2*tp/(2*tp+fp+fn), 0), 4),

Youden_J=round(youden_j, 4) # 修正后的Youden J值计算

)

}

\# ==================== 7. 获取测试集概率与评估 ====================

cat("\n正在计算训练集与测试集的评估指标...\n")

probs_test <- data.frame(sapply(names(base_models), function(m) get_probs(base_models[[m]], X_test)))

probs_test$Meta_Ensemble <- rowMeans(probs_test[, top3_models])

all_results <- data.frame()

for(m in model_names) {

all_results <- rbind(all_results, calc_metrics(y_train, probs_train_all[[m]], train_thresholds[[m]], m, "Train"))

all_results <- rbind(all_results, calc_metrics(y_test, probs_test[[m]], train_thresholds[[m]], m, "Test"))

}

cat("\n================ 模型性能评估表 ================\n")

print(all_results)

\# 修正：使用绝对路径写入CSV（替换为你的实际路径）

write.csv(all_results, file = "C:/BPPV修改/修改/Model_Performance_Metrics.csv", row.names = FALSE)

cat("结果已保存: C:/BPPV修改/修改/Model_Performance_Metrics.csv\n")

\# ==================== 8. DeLong检验 ====================

cat("\n正在执行DeLong检验 (测试集)...\n")

roc_list_test <- lapply(model_names, function(m) pROC::roc(y_test, probs_test[[m]], quiet=TRUE, levels=c(0,1), direction="<"))

names(roc_list_test) <- model_names

pval_matrix <- matrix(1, nrow=length(model_names), ncol=length(model_names), dimnames=list(model_names, model_names))

for(i in 1:(length(model_names)-1)) {

for(j in (i+1):length(model_names)) {

p_val <- as.numeric(pROC::roc.test(roc_list_test[[i]], roc_list_test[[j]], method="delong")$p.value)

pval_matrix[i, j] <- p_val

pval_matrix[j, i] <- p_val

}

}

df_pvals <- as.data.frame(round(pval_matrix, 4))

cat("\n================ DeLong检验P值矩阵 (测试集) ================\n")

print(df_pvals)

write.csv(df_pvals, "C:/BPPV修改/修改/DeLong_Test_P_Values.csv", row.names = FALSE)

cat("DeLong检验结果已保存: C:/BPPV修改/修改/DeLong_Test_P_Values.csv\n")

\# ==================== 9. 可视化绘图优化 ====================

cat("\n正在绘制高质量综合四合图...\n")

model_colors <- c("LR" = "#999999", "XGBoost" = "#E69F00", "SVM" = "#56B4E9",

"MLP" = "#009E73", "GBM" = "#0072B2", "Meta_Ensemble" = "#D55E00")

theme_pub <- theme_bw(base_size = 14) +

theme(

plot.title = element_text(size = 16, face = "bold", hjust = 0),

axis.title = element_text(size = 14, face = "bold"),

axis.text = element_text(size = 12, color = "black"),

panel.grid.minor = element_blank(),

panel.grid.major = element_line(linetype = "dotted", color = "grey80"),

legend.position = "right",

legend.title = element_text(size = 12, face = "bold"),

legend.text = element_text(size = 11),

legend.key.size = unit(0.5, "cm"),

strip.text = element_text(size = 12, face = "bold"),

plot.margin = margin(15, 15, 15, 15)

)

calc_nb <- function(y, p, ts) sapply(ts, function(pt) {

yp <- as.integer(p>=pt);

tp <- sum(y==1&yp==1); fp <- sum(y==0&yp==1);

if(pt==1) return(0);

(tp/length(y))-(fp/length(y))*(pt/(1-pt))

})

get_cal_data <- function(y, p, model, n_bins=10) {

valid <- !is.na(p) & !is.na(y)

y <- y[valid]; p <- p[valid]

q <- quantile(p, probs = seq(0, 1, length.out = n_bins + 1), na.rm = TRUE)

q <- unique(q)

if(length(q) < 2) return(NULL)

q[1] <- q[1] - 1e-5; q[length(q)] <- q[length(q)] + 1e-5

groups <- cut(p, breaks = q, labels = FALSE, include.lowest = TRUE)

data.frame(Pred = tapply(p, groups, mean), Obs = tapply(y, groups, mean), Model = model)

}

\# 通用绘图函数：所有模型四合图 (修复2: 修复括号缺失导致的语法错误)

plot_combined <- function(dataset_name, probs_data, y_true, roc_list, suffix) {

y_num <- as.numeric(unlist(y_true))

\# A: ROC曲线

roc_df <- do.call(rbind, lapply(model_names, function(m) {

r <- roc_list[[m]];

data.frame(FPR=1-r$specificities, TPR=r$sensitivities, Model=m)

}))

roc_labels <- sapply(model_names, function(m) {

r <- roc_list[[m]]

ci <- ci.auc(r)

sprintf("%s (%.3f, %.3f-%.3f)", m, auc(r), ci[1], ci[3])

})

p1 <- ggplot(roc_df, aes(FPR, TPR, color=Model)) +

geom_line(linewidth=1.2) +

geom_abline(linetype="dashed", color="grey60", linewidth=0.8) +

scale_color_manual(values = model_colors, labels = roc_labels) +

labs(title="A. ROC Curves", x="1 - Specificity", y="Sensitivity") +

theme_pub +

theme(legend.position=c(0.65, 0.15),

legend.title=element_blank(),

legend.background=element_rect(fill=alpha("white",0.9), color="grey80"))

\# B: PR曲线

pr_df <- do.call(rbind, lapply(model_names, function(m) {

p <- probs_data[[m]]; o <- order(p, decreasing=TRUE)

tp <- cumsum(y_num[o]==1); fp <- cumsum(y_num[o]==0)

data.frame(Recall=c(0, tp/sum(y_num==1)), Precision=c(1, tp/(tp+fp)), Model=m)

}))

pr_labels <- sapply(model_names, function(m) {

p <- probs_data[[m]]; o <- order(p, decreasing=TRUE)

tp <- cumsum(y_num[o]==1); fp <- cumsum(y_num[o]==0)

\# 修复: 补全缺失的右括号

pr_auc <- sum(diff(c(0, tp/sum(y_num==1))) * c(1, head(tp/(tp+fp),-1)))

sprintf("%s (%.3f)", m, pr_auc)

})

p2 <- ggplot(pr_df, aes(Recall, Precision, color=Model)) +

geom_line(linewidth=1.2) +

scale_color_manual(values = model_colors, labels = pr_labels) +

labs(title="B. PR Curves", x="Recall", y="Precision") +

theme_pub +

theme(legend.position=c(0.65, 0.85),

legend.title=element_blank(),

legend.background=element_rect(fill=alpha("white",0.9), color="grey80"))

\# C: 校准曲线

cal_df <- do.call(rbind, lapply(model_names, function(m) get_cal_data(y_num, probs_data[[m]], m)))

p3 <- ggplot(cal_df, aes(Pred, Obs, color=Model)) +

geom_abline(linetype="dashed", color="grey60", linewidth=0.8) +

geom_line(linewidth=1.2) +

geom_point(size=3) +

scale_color_manual(values = model_colors) +

labs(title="C. Calibration Curves", x="Mean Predicted Probability", y="Observed Fraction") +

theme_pub +

theme(legend.position="none")

\# D: DCA曲线

ts <- seq(0.01, 0.99, by=0.01); prev <- mean(y_num)

dca_df <- do.call(rbind, lapply(model_names, function(m) data.frame(Thresh=ts, NB=calc_nb(y_num, probs_data[[m]], ts), Model=m)))

dca_df <- rbind(dca_df, data.frame(Thresh=ts, NB=prev-(1-prev)*ts/(1-ts), Model="Treat All"))

dca_df <- rbind(dca_df, data.frame(Thresh=ts, NB=0, Model="Treat None"))

dca_df$Model <- factor(dca_df$Model, levels = c(model_names, "Treat All", "Treat None"))

p4 <- ggplot(dca_df, aes(Thresh, NB, color=Model, linetype=Model)) +

geom_hline(yintercept=0, linetype="dashed", color="grey50", linewidth=0.8) +

geom_line(linewidth=1.2) +

scale_color_manual(values = c(model_colors, "Treat All"="grey30", "Treat None"="grey50")) +

scale_linetype_manual(values = c(setNames(rep("solid", length(model_names)), model_names),

"Treat All"="dotted", "Treat None"="dashed")) +

coord_cartesian(xlim = c(0, 1), ylim = c(-0.1, 0.5)) +

labs(title="D. DCA Curves", x="Threshold Probability", y="Net Benefit") +

theme_pub +

theme(legend.position="none")

combined <- (p1 + p2) / (p3 + p4) +

plot_annotation(tag_levels = 'A', tag_suffix = '.') +

plot_layout(guides = "collect")

ggsave(paste0("C:/BPPV修改/修改/Combined_Plots_", suffix, ".png"), combined, width=16, height=12, dpi=300, units="in")

ggsave(paste0("C:/BPPV修改/修改/Combined_Plots_", suffix, ".tiff"), combined, width=16, height=12, dpi=300, units="in", compression="lzw")

cat(paste0("图表已保存: C:/BPPV修改/修改/Combined_Plots_", suffix, ".png / .tiff\n"))

}

roc_list_train <- lapply(model_names, function(m) pROC::roc(y_train, probs_train_all[[m]], quiet=TRUE, levels=c(0,1), direction="<"))

names(roc_list_train) <- model_names

plot_combined("Training Set", probs_train_all, y_train, roc_list_train, "Train")

plot_combined("Test Set", probs_test, y_test, roc_list_test, "Test")

\# ==================== 10. 最佳模型 单独四合图 ====================

cat("\n正在绘制最佳模型四合图...\n")

plot_best_model <- function(dataset_name, prob_data, y_true, roc_obj, suffix) {

y_num <- as.numeric(unlist(y_true))

roc_df <- data.frame(FPR = 1-roc_obj$specificities, TPR = roc_obj$sensitivities)

ci_obj <- ci.auc(roc_obj)

p1 <- ggplot(roc_df, aes(FPR, TPR)) +

geom_line(color="#D55E00", linewidth=1.5) +

geom_abline(linetype="dashed", color="grey60", linewidth=0.8) +

annotate("text", x=0.5, y=0.4, size=5, color="black",

label=sprintf("AUC = %.3f\n95%% CI: %.3f - %.3f", auc(roc_obj), ci_obj[1], ci_obj[3])) +

labs(title=paste0("A. ROC Curve (", dataset_name, ")"), x="1 - Specificity", y="Sensitivity") +

theme_pub

p <- prob_data; o <- order(p, decreasing=TRUE)

tp <- cumsum(y_num[o]==1); fp <- cumsum(y_num[o]==0)

pr_df <- data.frame(Recall=c(0, tp/sum(y_num==1)), Precision=c(1, tp/(tp+fp)))

pr_auc <- sum(diff(pr_df$Recall) * head(pr_df$Precision, -1))

p2 <- ggplot(pr_df, aes(Recall, Precision)) +

geom_line(color="#D55E00", linewidth=1.5) +

annotate("text", x=0.5, y=0.5, size=5, color="black", label=sprintf("PR-AUC = %.3f", pr_auc)) +

labs(title=paste0("B. PR Curve (", dataset_name, ")"), x="Recall", y="Precision") +

theme_pub

cal_df <- get_cal_data(y_num, prob_data, "Meta_Ensemble")

brier_val <- mean((y_num - prob_data)^2)

p3 <- ggplot(cal_df, aes(Pred, Obs)) +

geom_abline(linetype="dashed", color="grey60", linewidth=0.8) +

geom_line(color="#D55E00", linewidth=1.5) +

geom_point(color="#D55E00", size=3) +

annotate("text", x=0.2, y=0.8, size=5, color="black", label=sprintf("Brier = %.3f", brier_val)) +

labs(title=paste0("C. Calibration Curve (", dataset_name, ")"), x="Predicted Probability", y="Observed Probability") +

theme_pub

ts <- seq(0.01, 0.99, by=0.01); prev <- mean(y_num)

dca_df <- data.frame(Thresh=ts, NB=calc_nb(y_num, prob_data, ts), Model="Meta-Ensemble")

dca_df <- rbind(dca_df, data.frame(Thresh=ts, NB=prev-(1-prev)*ts/(1-ts), Model="Treat All"))

dca_df <- rbind(dca_df, data.frame(Thresh=ts, NB=0, Model="Treat None"))

dca_df$Model <- factor(dca_df$Model, levels = c("Meta-Ensemble", "Treat All", "Treat None"))

p4 <- ggplot(dca_df, aes(Thresh, NB, color=Model, linetype=Model)) +

geom_hline(yintercept=0, linetype="dashed", color="grey50", linewidth=0.8) +

geom_line(linewidth=1.2) +

scale_color_manual(values = c("Meta-Ensemble"="#D55E00", "Treat All"="grey30", "Treat None"="grey50")) +

scale_linetype_manual(values = c("Meta-Ensemble"="solid", "Treat All"="dotted", "Treat None"="dashed")) +

coord_cartesian(xlim = c(0, 1), ylim = c(-0.1, 0.5)) +

labs(title=paste0("D. DCA Curve (", dataset_name, ")"), x="Threshold Probability", y="Net Benefit") +

theme_pub +

theme(legend.position=c(0.7, 0.8), legend.title=element_blank())

combined <- (p1 + p2) / (p3 + p4) + plot_annotation(tag_levels = 'A', tag_suffix = '.')

ggsave(paste0("C:/BPPV修改/修改/Best_Model_", suffix, ".png"), combined, width=12, height=10, dpi=300, units="in")

ggsave(paste0("C:/BPPV修改/修改/Best_Model_", suffix, ".tiff"), combined, width=12, height=10, dpi=300, units="in", compression="lzw")

cat(paste0("图表已保存: C:/BPPV修改/修改/Best_Model_", suffix, ".png / .tiff\n"))

}

plot_best_model("Training Set", probs_train_all$Meta_Ensemble, y_train, roc_list_train$Meta_Ensemble, "Train")

plot_best_model("Test Set", probs_test$Meta_Ensemble, y_test, roc_list_test$Meta_Ensemble, "Test")

\# ==================== 11. SHAP解释（使用KernelSHAP彻底修复版） ====================

cat("\n正在生成SHAP解释图...\n")

\# 检查并安装 kernelshap 包

if (!requireNamespace("kernelshap", quietly = TRUE)) {

cat(" -> 正在安装缺失的包: kernelshap\n")

install.packages("kernelshap")

}

library(kernelshap)

\# 选择最佳模型 (Top 1 模型)

best_model <- base_models[[top3_models[1]]]

\# 1. 定义预测函数：必须接收两个参数

predict_prob <- function(model, X) {

as.numeric(predict(model, newdata = X, type = "prob")$Yes)

}

\# 2. 准备背景数据 (为了加速计算)

bg_X <- X_test[1:min(200, nrow(X_test)), , drop = FALSE]

cat("正在基于测试集计算SHAP值 (这可能需要几秒钟)...\n")

\# 3. 使用 KernelSHAP 计算模型无关的 SHAP 值

ks <- kernelshap(

object = best_model,

X = X_test,

bg_X = bg_X,

pred_fun = predict_prob

)

\# 4. 转换为 shapviz 对象进行可视化

shapviz_explainer <- shapviz(ks)

\# 生成 A. SHAP特征重要性

p_imp <- shapviz::sv_importance(shapviz_explainer, kind = "bar") +

theme_pub +

labs(title = "A. SHAP Feature Importance")

ggsave("C:/BPPV修改/修改/SHAP_Feature_Importance.png", plot = p_imp, width = 8, height = 6, dpi = 300)

\# 生成 B. SHAP Beeswarm plot (使用 kind = "beeswarm")

p_bee <- shapviz::sv_importance(shapviz_explainer, kind = "beeswarm") +

theme_pub +

labs(title = "B. SHAP Beeswarm Plot")

ggsave("C:/BPPV修改/修改/SHAP_Beeswarm_Plot.png", plot = p_bee, width = 10, height = 8, dpi = 300)

\# 生成特定样本的SHAP waterfall plot (ID345和ID365)

sample_ids <- c(345, 365)

for (sample_id in sample_ids) {

if(sample_id > nrow(X_test)) {

cat(sprintf("警告: ID %d 超出测试集样本范围，已跳过。\n", sample_id))

next

}

\# 获取该样本的真实预测概率

sample_prob <- predict_prob(best_model, X_test[sample_id, , drop = FALSE])

sample_true <- y_test[sample_id]

\# 绘制 waterfall 图形并赋值给变量

p_wf <- shapviz::sv_waterfall(shapviz_explainer, row_id = sample_id) +

theme_pub +

labs(title = sprintf("Waterfall Plot (ID: %d, Prob: %.2f)", sample_id, sample_prob))

\# 单独调用 ggsave 保存图形

ggsave(sprintf("C:/BPPV修改/修改/SHAP_Waterfall_%d.png", sample_id), plot = p_wf, width = 10, height = 8, dpi = 300)

cat(sprintf("SHAP waterfall plot for ID %d saved (Prob: %.2f, True: %d)\n",

sample_id, sample_prob, sample_true))

}

cat("\n====================================================\n")

cat("全部分析完成！\n")

cat("====================================================\n")

\# ==================== 12. 生成完整SHAP解释图（包含所有需求内容） ====================

cat("\n正在生成完整SHAP解释图（包含特征重要性、蜂群图、瀑布图及依赖关系图）...\n")

\# 确保shapviz和kernelshap包已加载

if (!requireNamespace("kernelshap", quietly = TRUE)) {

cat(" -> 正在安装缺失的包: kernelshap\n")

install.packages("kernelshap")

}

if (!requireNamespace("shapviz", quietly = TRUE)) {

cat(" -> 正在安装缺失的包: shapviz\n")

install.packages("shapviz")

}

library(kernelshap)

library(shapviz)

\# 选择最佳模型 (Top 1 模型)

best_model <- base_models[[top3_models[1]]]

\# 1. 定义预测函数：返回概率

predict_prob <- function(model, X) {

as.numeric(predict(model, newdata = X, type = "prob")$Yes)

}

\# 2. 准备背景数据

bg_X <- X_test[1:min(200, nrow(X_test)), , drop = FALSE]

cat("正在基于测试集计算SHAP值 (这可能需要几分钟)...\n")

\# 3. 使用 KernelSHAP 计算模型无关的 SHAP 值

ks <- kernelshap(

object = best_model,

X = X_test,

bg_X = bg_X,

pred_fun = predict_prob

)

\# 4. 转换为 shapviz 对象进行可视化

shapviz_explainer <- shapviz(ks)

\# ==================== 12.1 生成特征重要性条形图 ====================

p_imp <- shapviz::sv_importance(shapviz_explainer, kind = "bar") +

theme_pub +

labs(title = "A. SHAP Feature Importance") +

theme(legend.position = "none")

ggsave("C:/BPPV修改/修改/SHAP_Feature_Importance.png", plot = p_imp, width = 8, height = 6, dpi = 300)

\# ==================== 12.2 生成SHAP蜂群图 ====================

p_bee <- shapviz::sv_importance(shapviz_explainer, kind = "beeswarm") +

theme_pub +

labs(title = "B. SHAP Beeswarm Plot")

ggsave("C:/BPPV修改/修改/SHAP_Beeswarm_Plot.png", plot = p_bee, width = 10, height = 8, dpi = 300)

\# ==================== 12.3 生成瀑布图（显示概率） ====================

\# 计算基准概率和样本概率

baseline_prob <- mean(predict_prob(best_model, bg_X))

\# 指定需要提取的 ID (只保留345和365，删除1和2)

target_ids <- c(345, 365)

valid_indices <- c()

valid_ids <- c()

cat("正在尝试定位指定的 ID: 345 和 365...\n")

\# 辅助函数：在数据框的第一列或名为ID的列中查找目标ID

find_id_indices <- function(df, ids) {

id_col <- NULL

\# 优先检查第一列是否包含所有目标ID

if (all(ids %in% df[[1]])) {

id_col <- 1

} else {

\# 检查是否有名为 ID, Id, id 的列

id_cols <- intersect(c("ID", "Id", "id"), colnames(df))

if (length(id_cols) > 0) {

if (all(ids %in% df[[id_cols[1]]])) {

id_col <- id_cols[1]

}

}

}

if (!is.null(id_col)) {

indices <- c()

for (id in ids) {

\# 找到该 ID 在数据框中的行号

idx <- which(df[[id_col]] == id)[1]

indices <- c(indices, idx)

}

return(list(col = id_col, indices = indices))

}

return(NULL)

}

\# 1. 首先检查全局环境中是否存在包含 ID 的原始测试集数据框

\# （通常在划分数据时，X_test 是从原始 test_data 中提取特征列得到的）

found_result <- NULL

for (vname in ls(envir = .GlobalEnv)) {

obj <- get(vname, envir = .GlobalEnv)

if (is.data.frame(obj) && nrow(obj) == nrow(X_test)) {

result <- find_id_indices(obj, target_ids)

if (!is.null(result)) {

cat(sprintf("在全局变量 '%s' 的第 '%s' 列中找到目标 ID。\n", vname, result$col))

found_result <- result

break

}

}

}

if (!is.null(found_result)) {

\# 找到了原始数据框，且行数与 X_test 一致，认为顺序对应

valid_indices <- found_result$indices

valid_ids <- as.character(target_ids)

cat("成功映射 ID 到 X_test 的行索引：", valid_indices, "\n")

} else {

\# 2. 如果全局环境没找到，检查 X_test 的行名是否就是 ID

if (all(target_ids %in% as.numeric(rownames(X_test)))) {

for (id in target_ids) {

idx <- which(as.numeric(rownames(X_test)) == id)[1]

valid_indices <- c(valid_indices, idx)

valid_ids <- c(valid_ids, as.character(id))

}

cat("在 X_test 的行名中成功定位指定 ID。\n")

} else {

cat("警告: 无法在环境中自动定位包含 ID 345 和 365 的数据框。\n")

}

}

\# 如果经过上述努力仍未找到有效的行索引

if (length(valid_indices) < length(target_ids)) {

cat("为避免代码报错，将自动回退至选择 X_test 的前2个样本作为替代。\n")

valid_indices <- 1:min(2, nrow(X_test))

valid_ids <- as.character(valid_indices)

}

\# 遍历有效的索引生成瀑布图

for (i in seq_along(valid_indices)) {

idx <- valid_indices[i]

current_id <- valid_ids[i]

\# 获取该样本的真实预测概率

sample_prob <- predict_prob(best_model, X_test[idx, , drop = FALSE])

sample_true <- y_test[idx]

\# 生成瀑布图 - 使用 sv_waterfall

p_wf <- shapviz::sv_waterfall(

shapviz_explainer,

row_id = idx

) +

theme_pub +

labs(title = sprintf("C. Waterfall Plot (ID: %s, Prob: %.2f)", current_id, sample_prob)) +

theme(legend.position = "none")

\# 保存瀑布图

ggsave(sprintf("C:/BPPV修改/修改/SHAP_Waterfall_%s.png", current_id), plot = p_wf, width = 10, height = 8, dpi = 300)

cat(sprintf("SHAP waterfall plot for ID %s saved (Prob: %.2f, True: %d)\n",

current_id, sample_prob, sample_true))

}

\# ==================== 12.4 生成SHAP依赖关系散点图（4个特征） ====================

\# 从shapviz_explainer中获取SHAP值（关键修正：使用$S而不是shap_values）

shap_values <- shapviz_explainer$S

feature_data <- X_test

\# 创建四个特征的散点图

p_osteoporosis <- ggplot(data = feature_data, aes(x = osteoporosis, y = shap_values[, "osteoporosis"])) +

geom_point(alpha = 0.6, color = "#f46d43") +

labs(title = "Osteoporosis", x = "Osteoporosis", y = "SHAP value") +

theme_pub +

theme(plot.title = element_text(size = 14, face = "bold"))

p_calcium <- ggplot(data = feature_data, aes(x = calcium, y = shap_values[, "calcium"])) +

geom_point(alpha = 0.6, color = "#f46d43") +

labs(title = "Calcium", x = "Calcium", y = "SHAP value") +

theme_pub +

theme(plot.title = element_text(size = 14, face = "bold"))

p_vitamind <- ggplot(data = feature_data, aes(x = vitamind, y = shap_values[, "vitamind"])) +

geom_point(alpha = 0.6, color = "#f46d43") +

labs(title = "VitaminD", x = "VitaminD", y = "SHAP value") +

theme_pub +

theme(plot.title = element_text(size = 14, face = "bold"))

p_estradiol <- ggplot(data = feature_data, aes(x = estradiol, y = shap_values[, "estradiol"])) +

geom_point(alpha = 0.6, color = "#f46d43") +

labs(title = "Estradiol", x = "Estradiol", y = "SHAP value") +

theme_pub +

theme(plot.title = element_text(size = 14, face = "bold"))

\# 组合成四合图

p_dependence <- (p_osteoporosis + p_calcium) / (p_vitamind + p_estradiol) +

plot_annotation(tag_levels = 'A') +

plot_layout(guides = "collect", widths = c(1, 1), heights = c(1, 1))

\# 保存依赖关系图

ggsave("C:/BPPV修改/修改/SHAP_Dependence_All.png", plot = p_dependence, width = 16, height = 12, dpi = 300)

cat("\n====================================================\n")

cat("SHAP解释图生成完成！\n")

cat("生成的文件包括:\n")

cat(" - C:/BPPV修改/修改/SHAP_Feature_Importance.png (特征重要性条形图)\n")

cat(" - C:/BPPV修改/修改/SHAP_Beeswarm_Plot.png (SHAP蜂群图)\n")

cat(" - C:/BPPV修改/修改/SHAP_Waterfall_345.png (ID 345瀑布图)\n")

cat(" - C:/BPPV修改/修改/SHAP_Waterfall_365.png (ID 365瀑布图)\n")

cat(" - C:/BPPV修改/修改/SHAP_Dependence_All.png (4个特征的SHAP依赖关系图)\n")

cat("====================================================\n")
