# Supplementary material for: Prediction of benign paroxysmal positional vertigo recurrence in postmenopausal women: a machine learning-based clinical study
Source: Front Neurol. 2026 Aug 25;17:1890770. doi: 10.3389/fneur.2026.1890770 (PMC13549858; doi:10.3389/fneur.2026.1890770)
Supplement: Supplementary file 1 [file Data_Sheet_1.ZIP › supplementary materials/R Code(lasso+boruta).docx]

\# ==============================================================================

\# 机器学习特征选择与相关性分析 (使用 lambda.min 准则 + 容错保存)

\# ==============================================================================

\# 1. 加载必要的包

packages <- c("readxl", "dplyr", "glmnet", "Boruta", "corrplot")

new_packages <- packages[!(packages %in% installed.packages()[,"Package"])]

if(length(new_packages)) install.packages(new_packages)

library(readxl)

library(dplyr)

library(glmnet)

library(Boruta)

library(corrplot)

\# ==============================================================================

\# 第一步：数据读取与严格的数据预处理 (杜绝数据泄露)

\# ==============================================================================

data_train <- read_excel("训练集.xlsx", sheet = 1)

data_test <- read_excel("测试集.xlsx", sheet = 1)

\# 清洗列名

colnames(data_train) <- tolower(trimws(colnames(data_train)))

colnames(data_test) <- tolower(trimws(colnames(data_test)))

cat("清洗后的训练集列名：\n")

print(colnames(data_train))

if("id" %in% colnames(data_train)) data_train <- data_train %>% select(-id)

if("id" %in% colnames(data_test)) data_test <- data_test %>% select(-id)

if (!"type" %in% colnames(data_train)) stop("错误：数据中仍未找到 'type' 列！")

data_train$type <- as.factor(data_train$type)

data_test$type <- as.factor(data_test$type)

\# 缺失值填补：严格使用训练集的中位数填补两个集合

impute_values <- sapply(data_train, function(x) if(is.numeric(x)) median(x, na.rm = TRUE) else NA)

for (col in names(data_train)) {

if (is.numeric(data_train[[col]])) {

train_median <- impute_values[[col]]

data_train[[col]][is.na(data_train[[col]])] <- train_median

if (col %in% names(data_test)) data_test[[col]][is.na(data_test[[col]])] <- train_median

}

}

x_matrix <- as.matrix(data_train[, setdiff(names(data_train), "type")])

y_vector <- data_train$type

\# ==============================================================================

\# 第二步：LASSO回归分析与作图

\# ==============================================================================

set.seed(42)

cv_fit <- cv.glmnet(x_matrix, y_vector, family = "binomial", type.measure = "deviance", alpha = 1)

tiff("Figure1_LASSO_CV_Plot.tiff", width = 2000, height = 1800, res = 300)

par(mar = c(5, 5, 4, 2))

plot(cv_fit)

title("LASSO Cross-Validation (Selection of Optimal Lambda)", line = 2.5)

abline(v = log(cv_fit$lambda.min), col = "red", lty = 2, lwd = 1.5)

abline(v = log(cv_fit$lambda.1se), col = "blue", lty = 2, lwd = 1.5)

legend("topleft", legend = c("Min Lambda", "1SE Lambda"), col = c("red", "blue"), lty = 2)

dev.off()

fit_lasso <- glmnet(x_matrix, y_vector, family = "binomial", alpha = 1)

tiff("Figure2_LASSO_Coefficient_Path.tiff", width = 2000, height = 1800, res = 300)

par(mar = c(5, 5, 4, 2))

plot(fit_lasso, xvar = "lambda", label = TRUE, lwd = 1.5)

title("LASSO Coefficient Profiles", line = 2.5)

abline(v = log(cv_fit$lambda.min), col = "black", lty = 2, lwd = 2)

text(x = log(cv_fit$lambda.min), y = max(coef(fit_lasso))*0.9, labels = "Min Lambda", pos = 4)

dev.off()

\# 使用 lambda.min 准则提取变量

coef_matrix <- coef(cv_fit, s = "lambda.min")

lasso_vars <- rownames(coef_matrix)[which(as.numeric(coef_matrix) != 0)]

lasso_vars <- lasso_vars[lasso_vars != "(Intercept)"]

cat("\n================ LASSO 筛选结果 ================\n")

cat("使用 lambda.min 准则，LASSO实际筛选出的变量数:", length(lasso_vars), "\n")

cat("变量列表:", paste(lasso_vars, collapse = ", "), "\n")

cat("================================================\n")

\# ==============================================================================

\# 第三步：Boruta特征选择与作图

\# ==============================================================================

boruta_data <- data_train[, c(lasso_vars, "type")]

set.seed(42)

boruta_output <- Boruta(type ~ ., data = boruta_data, doTrace = 2, maxRuns = 500)

final_boruta <- TentativeRoughFix(boruta_output)

tiff("Figure3_Boruta_Importance_Boxplot.tiff", width = 2800, height = 2000, res = 300)

par(mar = c(10, 5, 4, 2))

plot(final_boruta, xlab = "", ylab = "Importance (Z-Score)", main = "Feature Importance Selection by Boruta", las = 2, cex.axis = 0.8)

legend("topleft", legend = c("Confirmed", "Tentative", "Rejected", "Shadow"), fill = c("green", "yellow", "red", "blue"), bty = "n", cex = 0.9)

dev.off()

final_vars <- getSelectedAttributes(final_boruta, withTentative = FALSE)

cat("\n================ Boruta 最终确认变量 ================\n")

print(final_vars)

\# 【增加容错机制】：如果文件被Excel占用，不至于程序崩溃

tryCatch({

write.csv(data.frame(Variables = final_vars), "Final_Model_Variables.csv", row.names = FALSE)

cat("最终变量已保存至 Final_Model_Variables.csv\n")

}, error = function(e) {

cat("\n【警告】无法保存CSV文件。可能是因为该文件正在被Excel或其他程序打开。\n")

cat("请关闭相关程序后重试，本次运行先直接在屏幕上输出变量：\n")

print(final_vars)

})

\# ==============================================================================

\# 第四步：保留最终变量的 Spearman 相关性分析

\# ==============================================================================

data_for_corr <- data_train[, final_vars]

cor_matrix <- cor(data_for_corr, method = "spearman", use = "complete.obs")

png("Figure4_Correlation_Heatmap.png", width = 1500, height = 1500, res = 170)

corrplot(cor_matrix, method = "color", type = "upper", order = "original",

addCoef.col = "black", number.cex = 0.8, tl.col = "black", tl.srt = 45,

tl.cex = 0.9, diag = TRUE,

col = colorRampPalette(c("blue", "white", "red"))(200),

title = "Spearman Correlation Matrix of Final Features", mar = c(0,0,2,0))

dev.off()

cat("\n所有R语言特征筛选与相关性分析已完成！\n")
