# Supplementary material for: Prediction of benign paroxysmal positional vertigo recurrence in postmenopausal women: a machine learning-based clinical study
Source: Front Neurol. 2026 Aug 25;17:1890770. doi: 10.3389/fneur.2026.1890770 (PMC13549858; doi:10.3389/fneur.2026.1890770)
Supplement: Supplementary file 1 [file Data_Sheet_1.ZIP › supplementary materials/Supplementary Table 1.docx]

Supplementary Table 1 Baseline characteristics of the training and validation sets

| Variable | Training set (n=289) | Validation set (n=123) | Test Statistic | P-value |
| --- | --- | --- | --- | --- |
| **Recurrence status** |  |  | χ² = 0.020 | 0.888 |
| No recurrence | 196 (67.8%) | 85 (69.1%) |  |  |
| Recurrence | 93 (32.2%) | 38 (30.9%) |  |  |
| **Age** | 59.10 ± 6.33 | 59.44 ± 4.78 | t = -0.527 | 0.599 |
| **Residence** |  |  | χ² = 3.628 | 0.057 |
| Rural | 77 (26.6%) | 45 (36.6%) |  |  |
| Urban | 212 (73.4%) | 78 (63.4%) |  |  |
| **Education** | 8 (6, 10) | 8 (6, 10) | Z = 0.072 | 0.942 |
| **BMI** | 27.32 ± 2.60 | 27.06 ± 2.60 | t = 0.928 | 0.354 |
| **Hypertension** |  |  | χ² = 0.661 | 0.416 |
| No | 208 (72.0%) | 94 (76.4%) |  |  |
| Yes | 81 (28.0%) | 29 (23.6%) |  |  |
| **Hyperlipidemia** |  |  | χ² = 2.010 | 0.156 |
| No | 225 (77.9%) | 87 (70.7%) |  |  |
| Yes | 64 (22.1%) | 36 (29.3%) |  |  |
| **Hyperglycemia** |  |  | χ² = 0.000 | 1.000 |
| No | 228 (78.9%) | 97 (78.9%) |  |  |
| Yes | 61 (21.1%) | 26 (21.1%) |  |  |
| **Migraine** |  |  | χ² = 1.087 | 0.297 |
| No | 185 (64.0%) | 86 (69.9%) |  |  |
| Yes | 104 (36.0%) | 37 (30.1%) |  |  |
| Cervical spondylosis |  |  | χ² = 0.691 | 0.406 |
| No | 233 (80.6%) | 94 (76.4%) |  |  |
| Yes | 56 (19.4%) | 29 (23.6%) |  |  |
| **Menieres disease** |  |  | χ² = 0.416 | 0.519 |
| No | 224 (77.5%) | 91 (74.0%) |  |  |
| Yes | 65 (22.5%) | 32 (26.0%) |  |  |
| **Osteoporosis** |  |  | χ² = 0.102 | 0.750 |
| No | 192 (66.4%) | 79 (64.2%) |  |  |
| Yes | 97 (33.6%) | 44 (35.8%) |  |  |
| **Otolith position** |  |  | χ² = 4.008 | 0.261 |
| Posterior SCC | 118 (40.8%) | 43 (35.0%) |  |  |
| Horizontal SCC | 75 (26.0%) | 40 (32.5%) |  |  |
| Anterior SCC | 55 (19.0%) | 28 (22.8%) |  |  |
| Mixed type | 41 (14.2%) | 12 (9.8%) |  |  |
| **DHI** |  |  | χ² = 3.745 | 0.154 |
| Mild | 110 (38.1%) | 59 (48.0%) |  |  |
| Moderate | 121 (41.9%) | 41 (33.3%) |  |  |
| Severe | 58 (20.1%) | 23 (18.7%) |  |  |
| **HADS-A** |  |  | χ² = 2.064 | 0.151 |
| Normal | 204 (70.6%) | 96 (78.0%) |  |  |
| Abnormal | 85 (29.4%) | 27 (22.0%) |  |  |
| **HADS-D** |  |  | χ² = 0.007 | 0.934 |
| Normal | 237 (82.0%) | 102 (82.9%) |  |  |
| Abnormal | 52 (18.0%) | 21 (17.1%) |  |  |
| **PSQI** |  |  | χ² = 0.030 | 0.862 |
| Normal | 224 (77.5%) | 97 (78.9%) |  |  |
| Abnormal | 65 (22.5%) | 26 (21.1%) |  |  |
| **VAS** | 5 (4, 6) | 4 (3, 5) | Z = 5.354 | <0.001 |
| **Calcium** | 2.34 (1.47, 3.01) | 2.37 (1.64, 2.95) | Z = 0.052 | 0.958 |
| **Vitamin D** | 19.38 (17.24, 21.25) | 19.71 (17.10, 21.52) | Z = 0.510 | 0.610 |
| **Estradiol** | 16.82 (14.07, 19.05) | 15.69 (14.15, 17.83) | Z = 1.951 | 0.051 |

BMI, Body Mass Index; DHI, Dizziness Handicap Inventory; HADS_A, Hospital Anxiety and Depression Scale - Anxiety subscale; HADS_D, Hospital Anxiety and Depression Scale - Depression subscale; PSQI, Pittsburgh Sleep Quality Index; VAS, Visual Analog Scale
